# Chromatin remodeling mediated by ARID1A is indispensable for normal hematopoiesis in mice

**DOI:** 10.1038/s41375-019-0438-4

**Published:** 2019-03-11

**Authors:** Lin Han, Vikas Madan, Anand Mayakonda, Pushkar Dakle, Teoh Weoi Woon, Pavithra Shyamsunder, Hazimah Binte Mohd Nordin, Zeya Cao, Janani Sundaresan, Ienglam Lei, Zhong Wang, H. Phillip Koeffler

**Affiliations:** 1 Cancer Science Institute of Singapore, National University of Singapore, Singapore, Singapore; 2 Department of Medicine, Yong Loo Lin School of Medicine, National University of Singapore, Singapore, Singapore; 3 Department of Cardiac Surgery, Cardiovascular Research Center, University of Michigan, Ann Arbor, MI USA; 4 Cedars-Sinai Medical Center, Division of Hematology/Oncology, UCLA School of Medicine, Los Angeles, CA USA; 5 Department of Hematology-Oncology, National University Cancer Institute of Singapore (NCIS), National University Hospital, Singapore, Singapore

**Keywords:** Haematopoietic stem cells, Haematopoiesis

## Abstract

Precise regulation of chromatin architecture is vital to physiological processes including hematopoiesis. ARID1A is a core component of the mammalian SWI/SNF complex, which is one of the ATP-dependent chromatin remodeling complexes. To uncover the role of ARID1A in hematopoietic development, we utilized hematopoietic cell-specific deletion of *Arid1a* in mice. We demonstrate that ARID1A is essential for maintaining the frequency and function of hematopoietic stem cells and its loss impairs the differentiation of both myeloid and lymphoid lineages. ARID1A deficiency led to a global reduction in open chromatin and ensuing transcriptional changes affected key genes involved in hematopoietic development. We also observed that silencing of ARID1A affected ATRA-induced differentiation of NB4 cells, suggesting its role in granulocytic differentiation of human leukemic cells. Overall, our study provides a comprehensive elucidation of the function of ARID1A in hematopoiesis and highlights the central role of ARID1A-containing SWI/SNF complex in maintaining chromatin dynamics in hematopoietic cells.

## Introduction

Hematopoiesis is a tightly regulated process that constantly generates enormous numbers of mature blood cells through a cascade of differentiation stages. Self-renewal, quiescence and differentiation of hematopoietic stem cells (HSC), as well as subsequent lineage choices into various blood cell types are precisely orchestrated by stage-specific transcriptional and epigenetic machineries. Chromatin state is transformed dynamically during differentiation [1] and epigenetic factors that remodel chromatin and facilitate establishment and maintenance of stage-specific pattern of gene expression are key to sustain normal hematopoietic development.

ARID1A (BAF250a) is a principal component of SWI/SNF (SWItch/Sucrose Non-Fermentable) family of evolutionary conserved, multi-subunit chromatin remodeling complexes. Rearrangements of chromatin structure mediated by SWI/SNF complexes are critical for modulation of gene expression, thereby, affecting a broad range of cellular processes, including proliferation and differentiation [2–5]. ARID1A resides exclusively in the BAF subclass of SWI/SNF remodelers and contains an ARID domain, which interacts with DNA in a sequence-nonspecific manner. Sequencing of cancer genomes has identified recurrent mutations of *ARID1A* in a wide range of tumors including gynecological, liver, gastric, and breast tumors [5–10]. *ARID1A* is the most frequently mutated member of the SWI/SNF family and high incidence of inactivating mutations in varied cancers along with emerging functional studies postulate ARID1A as a novel tumor suppressor [11–15]. Amongst hematological diseases, *ARID1A* is mutated in acute promyelocytic leukemia (APL) [16] and several lymphoid malignancies [17–26]. Nonetheless, the role of ARID1A in hematopoietic development and how its loss contributes to leukemogenesis remains elusive. Krosl et al. [27] observed increased frequency of fetal liver HSCs in ARID1A-deficient mouse embryos, possibly through regulation of the fetal liver microenvironment. However, perinatal lethality caused by constitutive deletion of ARID1A has precluded elucidation of its role in adult hematopoiesis.

In this study, we have employed hematopoietic-cell specific deletion of ARID1A in mice to address systematically its function in the hematopoietic development. We demonstrate that lack of ARID1A results in a range of hematopoietic defects including highly diminished reconstitution ability in transplantation models. Our results illustrate that ARID1A maintains frequency and quiescence of HSCs, and regulates differentiation of both myeloid and lymphoid lineages in a cell-intrinsic manner. Depletion of ARID1A leads to extensive decrease in chromatin accessibility including loss of open chromatin at promoter/enhancer regions of several key regulators of hematopoietic development.

## Materials and methods

### Generation of mice with hematopoietic specific deletion of *Arid1a*

Mice bearing floxed *Arid1a* allele (*Arid1a*^*f/f*^) in which exon 9 of *Arid1a* gene is flanked by loxP sites have been described before [28]. *Arid1a*^*f/f*^ mice were backcrossed for five generations to C57BL/6 mice before crossing with either Vav-iCre or Mx1-Cre transgenic strains to generate hematopoietic cell-specific deletion of *Arid1a*. In Vav-iCre model, either *Arid1a*^*f/f*^*;Vav-iCre*^*−*^ or *Arid1a*^*f/+*^*;Vav-iCre*^*−*^ sex-matched littermates were used as controls. To induce deletion of *Arid1a* in adult *Arid1a*^*f/f*^*;Mx1-Cre*^*+*^ mice (8–14 weeks old), five doses of 300 µg poly(I:C) (GE Healthcare) were injected intraperitoneally, every alternate day. Sex-matched *Arid1a*^*f/f*^*;Mx1-Cre*^*−*^ littermates were also administered poly(I:C) simultaneously and used as control in all experiments. All mice were maintained in the animal facility of Comparative Medicine Centre, National University of Singapore (NUS). All mice experiments were performed according to protocols approved by NUS Institutional Animal Care and Use Committee.

### Flow cytometry and FACS sorting

Stained cells were acquired on FACS LSR II flow cytometer (BD Biosciences) and sorted on FACSAria cell sorter (BD Biosciences). Data were analyzed using FACSDIVA software (BD Biosciences). Antibodies used for flow cytometry are listed in Supplementary Table 1.

### Competitive reconstitution assays

For competitive reconstitution assay with purified HSCs, we sorted LT-HSCs (CD34^−^Flt3^−^ LSK) four weeks after poly(I:C) injection from *Arid1a*^*f/f*^*;Mx1-Cre*^*+*^ and *Arid1a*^*f/f*^*;Mx1-Cre*^*−*^ mice. 150 LT-HSCs (CD45.2^+^) from either WT or *Arid1a* KO mice were mixed with 300,000 BM cells (CD45.1^+^) from Ptprca Pepcb/BoyJ (B6.SJL) competitor mice and injected intravenously into lethally irradiated (11 Gy) B6.SJL recipient mice. Reconstitution was assessed every four weeks in peripheral blood using flow cytometry. Blood leukocytes were stained with antibodies against CD3 (T cells), CD19 (B cells), CD11b, Gr1, F4/80 (granulocytes and monocytes) along with donor (CD45.2) and competitor (CD45.1) markers.

For assays with BM cells (prior to *Arid1a* deletion), CD45.2-expressing BM cells from either *Arid1a*^*f/f*^*;Mx1-Cre*^*+*^ or *Arid1a*^*f/f*^*;Mx1-Cre*^*−*^ mice were mixed in equal proportion with CD45.1-expressing (B6.SJL) competitor BM cells. Two million cells were injected into the tail vein of lethally irradiated B6.SJL recipient mice. Donor engraftment was assessed in peripheral blood 4 weeks after transplantation. Arid1a deletion was induced in recipient mice using poly(I:C) and reconstitution of different blood lineages was determined as described above.

### RNA-sequencing

cDNA libraries from FACS-sorted LT-HSC were prepared using SMART-Seq v4 Ultra Low Input RNA Kit (Clontech Laboratories). poly-A selected RNA from purified CMP, GMP, and MEP populations was used for library preparation using TruSeq RNA Sample Preparation Kit (Illumina) according to the manufacturer’s protocol.

Libraries were sequenced on HiSeq 4000 and 100 bp paired-end reads were aligned to murine reference transcriptome (GRCm38/mm10; Ensemble version 84) using Kallisto (version 0.43.1) [29]. Further details of RNA-seq data analysis are described in Supplementary Methods. Primers used for quantitative RT-PCR validation are listed in Supplementary Table 2.

### ATAC-sequencing

ATAC-seq libraries were prepared from Lin^−^Kit^+^ BM cell as previously described [30]. Transposed DNA libraries were sequenced on HiSeq 4000. Quality of the paired end 50 bp reads was assessed using fastqc v0.11.7 and reads were then aligned to the mm10 reference genome using Bowtie2 v2.3.4.1 with the parameters --very-sensitive -X 2000 --no-mixed --no-discordant [31]. PCR duplicate reads were removed using Picard MarkDuplicates v2.17.10 (http://broadinstitute.github.io/picard). The reads were filtered using samtools v1.5 for a minimum mapping quality of 10 and only properly paired reads were retained [32]. Additionally, any reads aligning to the mitochondrial chromosome were removed. Peaks were called subsequently using macs2 v2.1.1.20160309 with the parameters --keep-dup all -f BAMPE -q 0.05, while simultaneously generating bedgraph files in Reads Per Million scale [33]. Peaks were filtered against the ENCODE blacklist regions (https://sites.google.com/site/anshulkundaje/projects/blacklists) [34]. The bedgraph files were converted to bigWig file format using the bedGraphToBigWig v4 utility. Differential binding analysis was performed using DESeq2 v1.18.1 through the DiffBind v2.6.6 bioconductor package with an FDR cut-off of 0.01 [35, 36]. Heat maps were generated using the deeptools v3.0.2 package [37]. Peaks were annotated using homer annotatePeaks.pl script [38]. Genomic profile plots for ATAC-seq signals were generated using fluff v3.0.2 [39].

### Statistical analysis

Statistical analyses for all mice experiments were performed using GraphPad Prism 7 software.

### Accession codes

All sequencing data generated in this study were deposited in Gene Expression Omnibus database repository. The accession numbers are: GSE125846 and GSE125848 (RNA-seq), GSE125845 (ChIP-seq), and GSE125844 (ATAC-seq).

## Results

### Generation of hematopoietic cell-specific deletion of *Arid1a* in mice

Expression analysis in FACS-sorted populations from bone marrow (BM) and spleen of C57BL/6 mice showed that *Arid1a* was expressed ubiquitously across different hematopoietic lineages with relatively lower expression detected in mature myeloid cells (Supplementary Figure 1a).

**Fig. 1:**
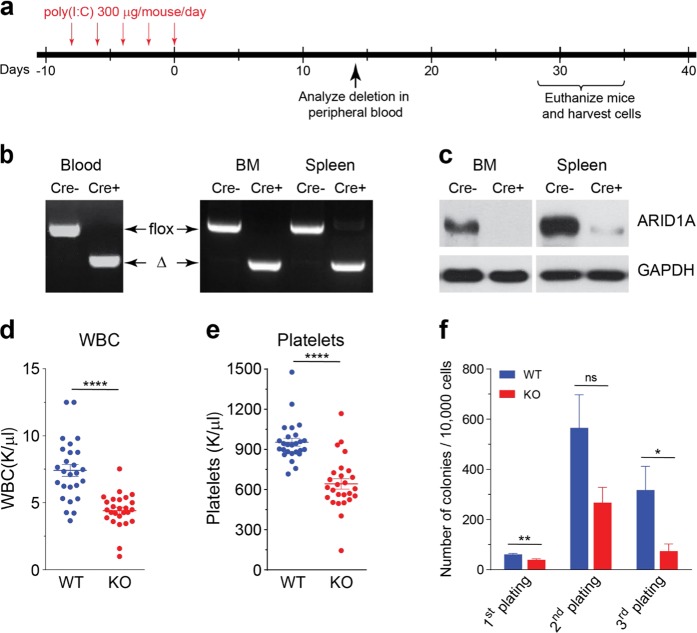
Hematopoietic-specific deletion of *Arid1a*. **a** Schematic of workflow to induce deletion of *Arid1a* in adult *Arid1a*^*f/f*^*;Mx1-Cre* mice using poly(I:C). **b** PCR analysis verifies poly(I:C)-induced deletion of *Arid1a* exon 9 in peripheral blood (2 weeks after poly(I:C) injection), BM and spleen (4 weeks after poly(I:C) injection) of *Arid1a*^*f/f*^*;Mx1-Cre*^*+*^ and *Arid1a*^*f/f*^*;Mx1-Cre*^*−*^ mice. Δ: deleted allele. **c** Western blot shows loss of ARID1A protein in BM and spleen of poly(I:C) treated *Arid1a*^*f/f*^*;Mx1-Cre* mice. **d**, **e** WBC (**d**) and platelet (**e**) counts in peripheral blood of *Arid1a*^*f/f*^*;Mx1-Cre*^*+*^ and *Arid1a*^*f/f*^*;Mx1-Cre*^*−*^ mice 4 weeks after poly(I:C) injection. **f** Number of colonies obtained in serial re-plating assay with total BM cells harvested from poly(I:C) treated *Arid1a*^*f/f*^*;Mx1-Cre* mice (*n* = 9 for first plating, *n* = 5 for second and third platings). Data are represented as mean ± SEM. *p < 0.05, **p < 0.01, ****p < 0.0001, ns = not significant

To analyze the function of ARID1A in hematopoietic development, mice carrying the *Arid1a* floxed allele (*Arid1a*^*f/f*^) [28] were crossed with either Vav-iCre [40] or Mx1-Cre [41] strains. We noted that *Arid1a*^*f/f*^;*Vav-iCre*^*+*^ mice were obtained at lower than expected frequency, both at birth (day 1) and weaning (day 21), suggesting perinatal mortality caused by *Arid1a* deletion in this model (Supplementary Table 3). Cre recombinase-mediated deletion of exon 9 and absence of ARID1A protein were verified in hematopoietic cells of the surviving *Arid1a*^*f/f*^;*Vav-iCre*^*+*^ mice (Supplementary Figure 1b–c). Owing to increased mortality of *Arid1a*^*f/f*^;*Vav-iCre*^*+*^ mice, more detailed analysis was performed simultaneously in mice where deletion of ARID1A was induced using the interferon inducible Mx1-Cre transgene. Administration of poly(I:C) caused rapid and complete deletion at *Arid1a* locus in peripheral blood and BM cells, while the deletion was ∼90% in spleen cells, which was also reflected in the residual protein levels observed in western blot analysis (Fig. 1a–c). Loss of ARID1A resulted in reduced numbers of WBCs and platelets in *Arid1a*^*f/f*^*;Mx1-Cre*^*+*^ mice compared to the *Arid1a*^*f/f*^*;Mx1-Cre*^*−*^ mice at 4 weeks after poly(I:C) treatment (Fig. 1d, e); and a similar trend was observed in *Arid1a*^*f/f*^;*Vav-iCre*^*+*^ mice (Supplementary Figure 1d-e). ARID1A-deficient BM cells exhibited decreased potential to generate myeloid colonies as both the number (Fig. 1f) and size (data not shown) of colonies was reduced in methylcellulose colony assays. Ex vivo deletion of floxed allele in Lin^−^Kit^+^ BM cells also resulted in fewer colonies (Supplementary Figure 1f-g). These initial observations suggested that depletion of ARID1A leads to perturbations in hematopoiesis.

### ARID1A maintains pool size and quiescence of HSCs

**Fig. 2:**
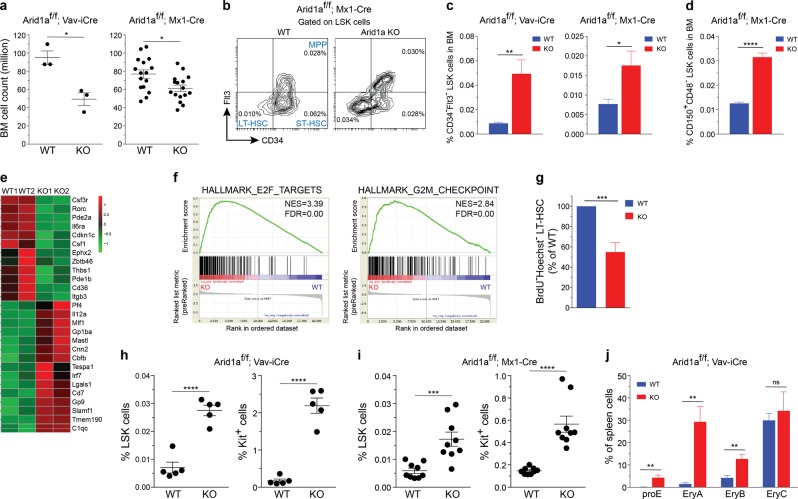
ARID1A is required to maintain HSC frequency. **a** BM cellularity in ARID1A-deficient mice compared with WT mice. Cell counts represent total leukocyte numbers in both femurs and tibias. For Vav-iCre model, mice were euthanized at 11–20 weeks of age; while for Mx1-Cre model, cellularity was determined 4 weeks after injection of poly(I:C). **b** Representative staining for LT-HSC, ST-HSC and MPP populations using CD34 and Flt3 antibodies in BM of *Arid1a*^*f/f*^*;Mx1-Cre*^*+*^ and *Arid1a*^*f/f*^*;Mx1-Cre*^*–*^ mice, four weeks after poly(I:C) injection. **c** Proportion of CD34^–^Flt3^–^ LSK cells in BM of *Arid1a* deficient and control mice (*n* = 5 for Vav-iCre; *n* = 6 for Mx1-Cre). **d** Percentage of CD150^+^CD48^−^ LSK cells in BM of *Arid1a*^*f/f*^*;Mx1-Cre*^*+*^ and *Arid1a*^*f/f*^*;Mx1-Cre*^*−*^ mice, four weeks after poly(I:C) injection (*n* = 4). **e** Heat map depicts differential expression of known regulators of hematopoiesis affected by loss of ARID1A in LTHSCs (CD34^−^Flt3^−^ LSK) compared to control (FDR < 0.1, except *Cdkn1c*, *Csf1*, *Cbfb*, where FDR < 0.25). LTHSC were sorted from the BM of *Arid1a*^*f/f*^*;Mx1-Cre*^*+*^ and *Arid1a*^*f/f*^*;Mx1-Cre*^*−*^ mice, 4 weeks after poly(I:C) injection. Genes involved in hematopoiesis were curated manually from Molecular Signatures Database v6.1 and GO datasets from AmiGO 2. **f** GSEA plots of cell cycle-related gene signatures, HALLMARK_E2F_TARGETS and HALLMARK_G2M_CHECKPOINT, in *Arid1a* KO vs control LTHSCs. NES: Normalized enrichment score. **g** Proportion of BrdU^−^Hoechst33342^−^ LTHSC in the BM of *Arid1a*^*f/f*^*;Mx1-Cre*^*+*^ and *Arid1a*^*f/f*^*;Mx1-Cre*^*−*^ mice (*n* = 6). Three weeks after administration of poly(I:C), mice were given BrdU in drinking water for one week before they were euthanized for experiment. (**h-i**) Proportion of LSK and Lin^–^Kit^+^ cells in the spleens of WT and *Arid1a* KO mice in Vav-iCre (**h**) and Mx1-Cre (**i**) models. **j** Frequencies of erythroid precursors in the BM of *Arid1a*^*f/f*^*;Vav-iCre*^*+*^ and *Arid1a*^*f/f*^*;Vav-iCre*^*–*^ mice. (proE: CD71^+^TER119^lo^; EryA: CD71^+^TER119^+^FSC^hi^; EryB: CD71^+^TER119^+^FSC^lo^ and EryC: CD71^−^TER119^+^FSC^lo^) (*n* = 5). Data are represented as mean ± SEM. *p < 0.05, **p < 0.01, ***p < 0.001, ****p < 0.0001, ns = not significant

We observed that Vav-iCre mediated deletion of *Arid1a* early in hematopoietic development resulted in markedly reduced number of BM leukocytes; and cellularity was also decreased significantly when *Arid1a* was deleted in adult mice using the Mx1-Cre model (Fig. 2a). In both models of ARID1A deficiency, the frequency and numbers of Lin^−^Sca1^+^Kit^+^ (LSK) cells, which contains HSCs, was not significantly altered (Supplementary Figure 2a–b). Nonetheless, the proportion and absolute numbers of long-term HSCs (LTHSCs), defined either as CD34^−^Flt3^−^LSK or CD150^+^CD48^−^LSK cells were elevated in BM from both *Arid1a*^*f/f*^*;Vav-iCre*^*+*^ and *Arid1a*^*f/f*^*;Mx1-Cre*^*+*^ mice (4 weeks after poly(I:C) injection), compared with the control mice (Fig. 2b–d; Supplementary Figure 2c–f). We observed that loss of one *Arid1a* allele also resulted in increased percentage of LT-HSCs in bone marrow (Supplementary Figure 2g), suggesting a dose-dependent effect of ARID1A in maintaining HSC frequency. Global transcriptome analysis of LT-HSCs from wildtype (WT) and ARID1A-deficient BM revealed dysregulated expression of genes crucial for hematopoietic development including *Csf3r*, *Il6ra*, *Csf1*, and *Cbfb* (Fig. 2e; Supplementary Figure 2h and Supplementary Table 4). GSEA identified that genesets associated with cell cycle were significantly enriched in *Arid1a* KO cells compared with the WT cells (Fig. 2f). In vivo BrdU incorporation assays showed that the proportion of non-cycling HSCs (BrdU^−^Hoechst33342^−^LTHSCs) was significantly reduced in ARID1A-deficient BM, indicating loss of quiescence (Fig. 2g).

Flow cytometric analysis of ARID1A-deficient spleens in both Vav-iCre and Mx1-Cre models demonstrated an expansion of progenitor/stem cell populations compared with the WT controls. This was evident from higher frequency and absolute number of Lin^−^Kit^+^ and LSK cells in the spleen of ARID1A-deficient mice (Fig. 2h, i; Supplementary Figure 3a-d). We also observed a marked elevation in erythroid precursors in spleen of *Arid1a*^*f/f*^*;Vav-iCre*^*+*^ mice, although the increase was less significant in spleens of Mx1-Cre^+^ mice four weeks after poly(I:C) injection (Fig. 2j; Supplementary Figure 3e), indicating onset of extramedullary hematopoiesis.

### *Arid1a* deletion leads to defects in development of myeloid and erythroid cells

**Fig. 3:**
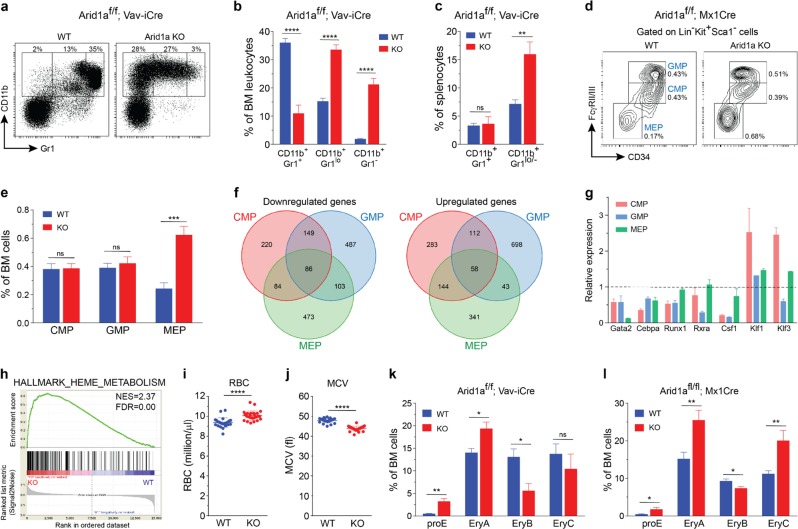
Defects in myeloid differentiation in *Arid1a* knockout mice. **a** Representative FACS profiles for BM cells from *Arid1a*^*f/f*^*;Vav-iCre*^*+*^ and control mice stained with CD11b and Gr1 antibodies. **b** Frequencies of myeloid populations based on staining for Gr1 and CD11b markers as depicted in (**a**) (*n* = 5). **c** Proportion of CD11b^+^Gr1^+^ and CD11b^+^Gr1^lo/−^ myeloid cells in the spleen of WT and *Arid1a* KO (Vav-iCre) mice (*n* = 5). **d** FACS plots depict representative staining of CMP, GMP and MEP cells in BM of poly(I:C) treated *Arid1a*^*f/f*^*;Mx1-Cre*^*+*^ and *Arid1a*^*f/f*^*;Mx1-Cre*^*−*^ mice. **e** Percentages of CMP, GMP, and MEP cells in the BM of WT and *Arid1a* KO (Mx1-Cre) mice (*n* = 6). **f** Venn diagrams depict overlap of upregulated and downregulated genes amongst CMP, GMP and MEP cells (FDR < 0.1, mean expression > 1 & absolute log_2_ fold change > 0.5). RNA-sequencing was performed on cells sorted from *Arid1a*^*f/f*^*;Mx1-Cre*^*+*^ and *Arid1a*^*f/f*^*;Mx1-Cre*^*−*^ mice 4 weeks after poly(I:C) treatment. **g** Quantitative RT-PCR analysis of selected genes differentially expressed (RNA-seq) in at least one of the three populations (CMP, GMP, and MEP) in the *Arid1a* KO mice compared with the WT mice. Cells were sorted from *Arid1a*^*f/f*^*;Mx1-Cre*^*+*^ and *Arid1a*^*f/f*^*;Mx1-Cre*^*−*^ mice four weeks after poly(I:C) injection. Bars represent relative expression in *Arid1a* KO cells compared to WT cells, which were assigned a value of 1 (depicted by a dashed line), for every gene in each cell population. Results are cumulative of two independent experiments. Transcript levels of *β-actin* were used for normalization. **h** GSEA plot for geneset HALLMARK_HEME_METABOLISM in the comparison of WT and ARID1A*-*deficient CMP cells. NES: Normalized enrichment score. **i**, **j** Number of RBC (**i**) and mean corpuscular volume (MCV) (**j**) in peripheral blood of poly(I:C) treated *Arid1a*^*f/f*^*;Mx1-Cre*^*+*^ and *Arid1a*^*f/f*^*;Mx1-Cre*^*−*^ mice. **k**, **l** Frequency of erythroid precursors (proE: CD71^+^TER119^lo^; EryA: CD71^+^TER119^+^FSC^hi^; EryB: CD71^+^TER119^+^FSC^lo^ and EryC: CD71^−^TER119^+^FSC^lo^) in BM of *Arid1a*^*f/f*^*;Vav-iCre* (**k**) (*n* = 5) and *Arid1a*^*f/f*^*;Mx1-Cre* (**l**) (*n* = 6) mice. Results represent mean ± SEM. *p < 0.05, **p < 0.01, ***p < 0.001, ****p < 0.0001, ns = not significant

To understand how ARID1A regulates production of mature blood cells, we carefully analyzed differentiation towards myeloid and lymphoid lineages in ARID1A-deficient mice. ARID1A deficiency caused defects in granulocytic differentiation in BM of both Vav-iCre and Mx1-Cre models. This was evident by increased frequency of immature granulocytes/monocytes (CD11b^+^Gr1^lo^ and CD11b^+^Gr1^−^) and a decrease in proportion of mature granulocytes (CD11b^+^Gr1^+^) in both deletion models (Fig. 3a, b; Supplementary Figure 4a–b). An increase in immature granulocytes/monocytes was also observed in the spleens of *Arid1a* KO mice, although the proportion of mature granulocytes was unaffected (Fig. 3c; Supplementary Figure 4c). Further analyses of myeloid progenitors in the BM of poly(I:C) treated *Arid1a*^*f/f*^*;Mx1-Cre*^*+*^ and *Arid1a*^*f/f*^*;Mx1-Cre*^*−*^ mice revealed that the proportion of common myeloid precursors (CMP; Lin^−^Kit^+^Sca1^−^CD34^+^FcγRII/III^lo^) and granulocyte monocyte precursors (GMP; Lin^−^Kit^+^Sca1^−^CD34^+^FcγRII/III^hi^) were unchanged in ARID1A-deficient BM; however, these populations exhibited reduced surface expression of CD34 compared with the control mice (Fig. 3d, e). Interestingly, an elevated frequency of megakaryocyte erythrocyte progenitors (MEP; Lin^−^Kit^+^Sca1^−^CD34^−^FcγRII/III^−^) was observed in the BM of *Arid1a*^*f/f*^*;Mx1-Cre*^*+*^ mice (Fig. 3d, e). To determine the molecular basis of the observed defects in myeloid differentiation, gene expression changes were analyzed using RNA-sequencing in sorted CMP, GMP, and MEP cells isolated 4 weeks after poly(I:C) injection from *Arid1a*^*f/f*^*;Mx1-Cre*^*+*^ and *Arid1a*^*f/f*^*;Mx1-Cre*^−^ mice. Complete deletion of *Arid1a* exon 9 was evident in cells from poly(I:C) treated *Arid1a*^*f/f*^*;Mx1-Cre*^*+*^ mice (Supplementary Figure 4d). Loss of ARID1A resulted in dysregulated expression of several genes either across all three populations or in a stage-specific manner (Fig. 3f; Supplementary Table 5). Importantly, altered gene expression in CMP, GMP, and MEP cells correlated well with changes in histone modifications (H3K27ac and H3K4me3) associated with transcriptional activation at respective loci (Supplementary Figure 4e-f). We used quantitative RT-PCR to validate altered expression of genes crucial for hematopoietic differentiation including *Gata2*, *Cebpa*, *Runx1*, *Rxra*, *Csf1*, *Klf1,* and *Klf3* (Fig. 3g). GSEA uncovered upregulation of genes involved in metabolism of heme and erythroblast differentiation in KO CMP and MEP cells (Fig. 3h; Supplementary Figure 5a), indicating importance of ARID1A in erythropoiesis. We observed that the RBCs displayed microcytosis in peripheral blood and BM of ARID1A-deficient mice (both Mx1-Cre and Vav-iCre models) (Fig. 3i, j; Supplementary Figure 5b–d). Further examination of erythroid differentiation in BM using surface expression of CD71 and TER119 showed an accumulation of proerythroblasts (CD71^+^TER119^lo^) and altered frequencies of subsequent stages of erythrocyte maturation in the BM of mice lacking ARID1A (Fig. 3k, l; Supplementary Figure 5e–f**)**. Collectively, these data demonstrate that ARID1A plays a pivotal role in myeloid and erythroid differentiation.

### Impaired lymphopoiesis in ARID1A-deficient mice

**Fig. 4:**
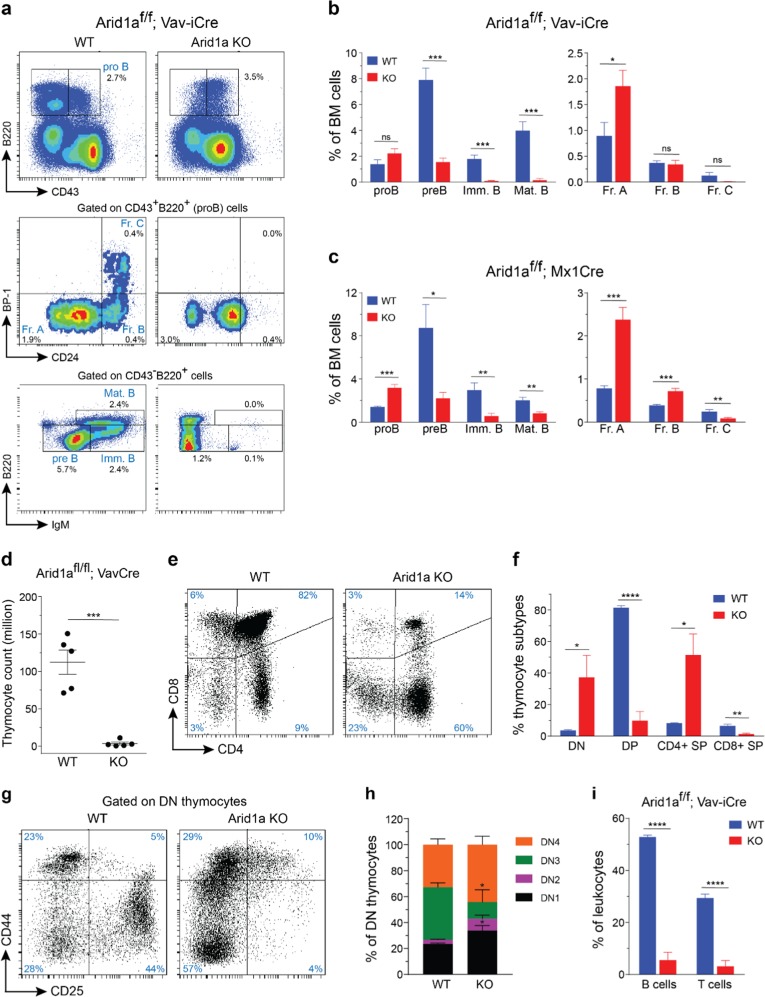
*Arid1a* knockout impairs lymphoid development. **a** Representative FACS plots show maturational stages of B cells in the BM of *Arid1a*^*f/f*^*;Vav-iCre*^*+*^ and control mice. **b**, **c** Proportion of cells in different B-cell developmental stages in the BM of WT and *Arid1a* KO [Vav-iCre (**b**) (*n* = 5) and Mx1-Cre (**c**) (*n* = 6)] mice. proB: CD43^+^B220^+^, preB: CD43^−^B220^+^IgM^−^, immature B (Imm. B): CD43^−^B220^+^IgM^+^, mature B (Mat. B): CD43^−^B220^hi^IgM^+^, Fraction A (Fr. A): CD24^−^BP1^−^ proB cells, Fraction B (Fr. B): CD24^+^BP1^−^ proB cells, Fraction C (Fr. C): CD24^+^BP1^+^ proB cells. **d** Thymus cellularity in *Arid1a*^*f/f*^*;Vav-iCre*^*+*^ and control (either *Arid1a*^*f/f*^*;Vav-iCre*^*−*^ or *Arid1a*^*f/+*^*;Vav-iCre*^*−*^ littermates) mice (11–20 weeks). **e** FACS plots depict representative staining of four major thymic populations: DN (CD4^−^CD8^−^), DP (CD4^+^CD8^+^), CD4^+^ and CD8^+^ SP cells in the thymus of *Arid1a*^*f/f*^*;Vav-iCre*^*+*^ and control mice. **f** Frequencies of DP, DN, CD4^+^ SP and CD8^+^ SP populations in *Arid1a*^*f/f*^*;Vav-iCre*^*+*^ and control mice (*n* = 5). **g** Representative flow cytometry staining for CD44 and CD25 expression within the DN population in the thymus of *Arid1a* KO (Vav-iCre) and control mice. **h** Proportions of DN1 (CD44^+^CD25^−^ DN), DN2 (CD44^+^CD25^+^ DN), DN3 (CD44^−^CD25^+^ DN) and DN4 (CD44^−^CD25^−^ DN) sub-populations within the DN compartment of thymus of *Arid1a*^*f/f*^*;Vav-iCre*^*+*^ and control mice (*n* = 5). **i** Frequencies of B cells (CD19^+^) and T cells (CD3^+^) in the spleen of WT and *Arid1a* KO mice (*n* = 5). Data are represented as mean ± SEM. *p < 0.05, **p < 0.01, ***p < 0.001, ****p < 0.0001, ns = not significant

Next, we assessed the effect of ARID1A loss on the lymphoid compartment. Flow cytometric analysis revealed that the proportion of proB cells (CD43^+^B220^+^), was increased in *Arid1a* KO BM, in both Vav-iCre and Mx1-Cre models (Fig. 4a–c). Further fractionation of proB cells using a scheme described before [42] demonstrated a severe block in differentiation from Fraction A (CD24^−^BP1^−^) to Fraction B (CD24^+^BP1^−^) proB cells (Fig. 4a–c). This resulted in significantly lower proportions of preB (CD43^−^IgM^−^B220^+^), immature B (CD43^−^IgM^+^B220^+^), and mature B (CD43^−^IgM^+^B220^hi^) cells in ARID1A-deficient BM (Fig. 4a–c).

Next, we assessed T-cell development in mice lacking ARID1A. Vav-iCre mediated deletion of *Arid1a* led to thymic atrophy (Fig. 4d) accompanied by severe perturbation in major T-cell compartments. CD4^−^CD8^−^ double negative (DN) compartment comprises of immature thymocyte populations, and immediately precedes the CD4^+^CD8^+^ double positive (DP) stage in development. We observed a striking reduction in DP cells with a concomitant increase in the proportion of DN and CD4^+^ SP cells in the thymi of *Arid1a*^*f/f*^;*Vav-iCre*^*+*^ mice compared with the control mice (Fig. 4e, f). Within the DN compartment, the proportions of DN1 (CD44^+^CD25^−^) and DN2 (CD44^+^CD25^+^) cells were elevated, while DN3 (CD44^−^CD25^+^) subpopulation was reduced in KO thymi, suggesting early arrest of thymocyte development (Fig. 4g, h).

We noted that Mx1-Cre mediated deletion of ARID1A was incomplete in thymoctyes 4 weeks after administration of poly(I:C) (Supplementary Figure 6a–b). Nonetheless, partial loss of ARID1A in this model caused rapid decline in thymic cellularity (Supplementary Figure 6c) and a trend towards altered frequencies of major thymic subsets (along the lines of the phenotype in the Vav-iCre deletion model) was noted (Supplementary Figure 6d-e). PCR analysis of sorted thymocyte subsets revealed a nearly complete deletion of *Arid1a* floxed allele in the DN fraction while the recombination was partial in DP and SP populations (Supplementary Figure 6f). These data demonstrate that ARID1A is required for normal T cell maturation in mice.

Defective maturation of the lymphoid cells led to substantially lower proportions of mature B and T cells in the spleens of ARID1A-deficient mice (Fig. 4i; Supplementary Figure 6g). Overall, our experiments establish a vital role of ARID1A in lymphoid differentiation.

### ARID1A is essential for HSC function

**Fig. 5:**
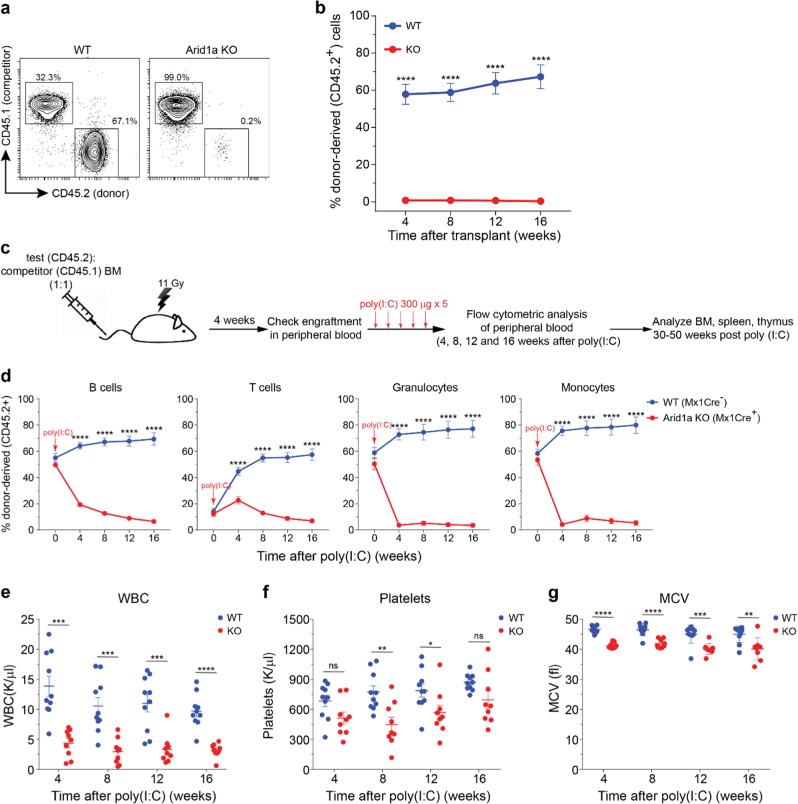
ARID1A is essential for reconstitution ability of HSCs. **a** Representative plots show donor chimerism (CD45.2) in peripheral blood of recipients transplanted with LTHSCs (CD34^−^FLT3^−^ LSK) from either control or *Arid1a* KO (Mx1-Cre) mice in competitive repopulation assay. **b** Average donor chimerism in recipient mice transplanted as in (**a**) and measured at 4, 8, 12, and 16 weeks post transplantation. Data are cumulative of two independent transplantation experiments (nine recipients/genotype). **c** Experimental design for competitive repopulation assay using total BM cells (prior to deletion of *Arid1a*). Lethally irradiated recipients were transplanted with BM cells (either *Arid1a*^*f/f*^*;Mx1-Cre*^*+*^ or *Arid1a*^*f/f*^*;Mx1-Cre*^*−*^ BM mixed in equal proportion with competitor BM) and deletion of *Arid1a* was induced four weeks post transplantation using poly(I:C). **d** Frequency of donor-derived B cells, T cells, granulocytes and monocytes in peripheral blood of recipient mice at different time points post poly(I:C) administration. Two transplant experiments were performed as illustrated in (**c**) (11 recipients/genotype). **e**–**g** Non-competitive transplant assays. BM cells from either *Arid1a*^*f/f*^*;Mx1-Cre*^*+*^ or *Arid1a*^*f/f*^*;Mx1-Cre*^*−*^ mice were injected into lethally irradiated mice and *Arid1a* deletion was induced after 4 weeks. WBC count (**e**), platelet count (**f**) and MCV (**g**) were determined every four weeks after poly(I:C) injection. Two independent transplantation experiments are summarised (WT = 10 recipients, *Arid1a* KO = 9 recipients). Data are represented as mean ± SEM. *p < 0.05, **p < 0.01, ***p < 0.001, ****p < 0.0001, ns = not significant

To assess the functional activity of ARID1A-deficient HSCs, FACS-sorted LTHSCs from either poly(I:C)-treated *Arid1a*^*f/f*^*;Mx1-Cre*^*+*^ or *Arid1a*^*f/f*^*;Mx1-Cre*^*−*^ mice were transplanted in competitive repopulation assays. We detected a striking loss of donor contribution in peripheral blood of recipient mice transplanted with *Arid1a* KO cells compared with WT cells (Fig. 5a, b; Supplementary Figure 7a). Proportion of donor-derived cells was markedly decreased in BM and other lymphoid organs of the recipients 16–25 weeks post-transplantation (Supplementary Figure 7b-d). This illustrated that despite an increase in frequency of the phenotypically defined HSC population, ARID1A deficiency severely impaired their reconstitution ability. To verify that ARID1A deficiency did not impede homing of the HSCs to the hematopoietic niche, ARID1A was deleted only after establishment of BM chimeras from *Arid1a*^*f/f*^*;Mx1-Cre*^*+*^ and *Arid1a*^*f/f*^*;Mx1-Cre*^−^ mice (Fig. 5c). Four weeks after transplantation, we detected a similar engraftment ability of BM cells from *Arid1a*^*f/f*^*;Mx1-Cre*^*+*^ and *Arid1a*^*f/f*^*;Mx1-Cre*^−^ mice (Supplementary Figure 8a). ARID1A deletion following administration of poly(I:C) resulted in rapid decline of donor-derived cells in peripheral blood of Mx1-Cre^+^ mice compared with the Mx1-Cre^−^ mice (Fig. 5d). Deficiency of ARID1A affected repopulation of both lymphoid and myeloid cells in the peripheral blood (Fig. 5d). Similarly, donor-chimerism was markedly reduced in stem/progenitor population in the BM and mature blood cells in spleen and thymus of recipients transplanted with *Arid1a* KO cells (Supplementary Figure 8b-d). Poor reconstitution ability of *Arid1a* KO cells was also evident in competitive repopulation assays using BM cells from *Arid1a*^*f/f*^*;Vav-iCre*^*+*^ mice compared with the control mice (Supplementary Figure 8e). To verify further cell-intrinsic effect of ARID1A deficiency on HSC function, non-competitive repopulation assays were performed. ARID1A was deleted in recipient mice four weeks after transplantation of BM from either *Arid1a*^*f/f*^*;Mx1-Cre*^*+*^ or *Arid1a*^*f/f*^*;Mx1-Cre*^−^ mice. Loss of ARID1A resulted in reduction in numbers of WBCs and platelets along with emergence of smaller RBCs in the peripheral blood (Fig. 5e–g; Supplementary Figure 8f), thus recapitulating the phenotype observed in the *Arid1a*^*f/f*^*;Mx1-Cre*^*+*^ mice. Collectively, these data demonstrate that ARID1A is vital for the repopulation ability of HSCs and its deficiency results in a competitive disadvantage.

### ARID1A regulates chromatin accessibility at loci critical for hematopoiesis

To assess how ARID1A-containing SWI/SNF complex regulates chromatin accessibility in myeloid precursors, Assay for Transposase Accessible Chromatin with high-throughput sequencing (ATAC-seq) was performed on sorted Lin^−^Kit^+^ BM cells from poly(I:C) treated *Arid1a*^*f/f*^*;Mx1-Cre*^*+*^ and *Arid1a*^*f/f*^*;Mx1-Cre*^−^ mice. We observed that the biological replicates for WT and *Arid1a* KO samples were highly concordant (Supplementary Figure 9a-b). ATAC-seq peaks localized predominantly to intergenic and intronic regions but were also present around transcriptional start sites (TSS)/promoter regions (Supplementary Figure 9c). Integrative analysis of ATAC-seq peaks with ChIP-seq data showed that open chromatin regions were enriched within both promoter (H3K4me3) and enhancer (H3K27ac) histone marks (Supplementary Figure 9d).

**Fig. 6:**
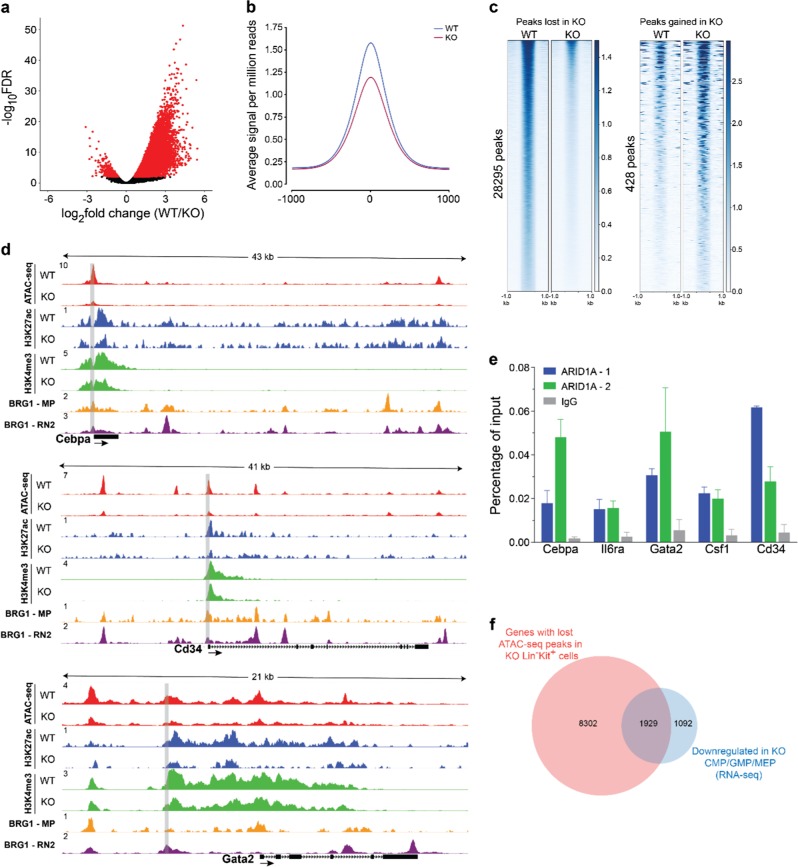
ARID1A maintains chromatin accessibility in hematopoietic cells. **a** Volcano plot shows ATAC-seq peaks identified in WT and *Arid1a* KO Lin^−^Kit^+^ BM cells. Cells were sorted from *Arid1a*^*f/f*^*;Mx1-Cre*^*+*^ and *Arid1a*^*f/f*^*;Mx1-Cre*^*−*^ mice four weeks after administration of poly(I:C). Peaks enriched significantly in either WT or *Arid1a* KO cells (FDR < 0.01) are depicted in red. **b** Average signal for all ATAC-seq peaks. **c** Heat maps show ATAC-seq peaks (scaled to 1 Kb) identified as either significantly closed (left) or gained (right) in KO cells using DiffBind analysis package. **d** Representative tracks for ATAC-seq signal (Lin^−^Kit^+^ BM cells), H3K27ac and H3K4me3 marks (both Lin^−^ BM cells) and BRG1 ChIP-seq (macrophages (GSM2663828) and RN2 cells (GSM2092897)) at *Cebpa*, *Cd34* and *Gata2* loci. Gray rectangles encompass the region analysed for binding of ARID1A using ChIP-qPCR. **e** ChIP-qPCR analysis of ARID1A occupancy at the loci of *Cebpa*, *Cd34*, *Il6ra*, *Csf1* and *Gata2* genes in 32D cells using two different ARID1A antibodies (ARID1A-1: Abcam ab182560; ARID1A-2: GeneTex GTX129433). Enrichment was calculated as percentages of input. Error bars represent SEM for two independent ChIP experiments for each locus. **f** Venn diagram shows overlap of genes with significantly reduced ATAC-seq signal in KO Lin^−^Kit^+^ BM cells and genes downregulated in *Arid1a* KO CMP, GMP, and MEP cells (RNA-seq) compared with the WT cells

We observed reduced number of ATAC-seq peaks in *Arid1a*-deficient cells, suggesting global loss of open chromatin in KO cells compared with the WT cells (Fig. 6a, b; Supplementary Figure 9c). We performed differential analysis using DiffBind [36], which revealed significantly reduced signal at 28,295 loci in KO cells, while 428 peaks were gained in KO cells compared with the WT cells, thus indicating reduced chromatin accessibility upon loss of ARID1A in Lin^−^Kit^+^ BM cells (Fig. 6c; Supplementary Figure 9e and Supplementary Table 6). Peaks differentially enriched upon deletion of *Arid1a* were located primarily in the intergenic and intronic regions (Supplementary Figure 9f-g). Importantly, significant reduction of ATAC-seq signal occurred in *Arid1a* KO cells at several key loci, which harbor genes involved in hematopoiesis, including *Cebpa*, *Cd34*, *Csf1*, *IL6ra* and *Gata2* (Fig. 6d and Supplementary Figure 10). Chromatin occupancy of both H3K27ac and H3K4me3, which mark transcriptionally active sites, was also reduced in *Arid1a*-deficient Lin^−^ cells at these loci (Fig. 6d and Supplementary Figure 10), demonstrating a correlation between chromatin accessibility and epigenetic signatures. To assess further if ARID1A directly binds to and regulates key genes involved in hematopoiesis, we performed ChIP-qPCR analysis using two anti-ARID1A antibodies. We observed an enrichment of ARID1A binding at promoter/putative regulatory elements of all the five genes with both ARID1A antibodies compared to the IgG control (Fig. 6e). Importantly, analysis of available ChIP-seq data for BRG1 [43, 44], a catalytic subunit of SWI/SNF complex, in mouse macrophages and RN2 cells (murine MLL-AF9/NrasG12D AML cells) showed extensive overlap of its occupancy with open chromatin regions at *Cebpa*, *Cd34*, *Csf1*, *IL6ra*, and *Gata2* loci (Fig. 6d and Supplementary Figure 10), thus substantiating SWI/SNF complex-mediated regulation of chromatin architecture at these loci. We noted that top four motifs identified in ATAC-seq peaks lost in *Arid1a* KO cells showed overlap with DNA sequences recognized by PU.1, RUNX1, GATA, and CEBPA (Supplementary Figure 9h).

Next, we determined if altered chromatin accessibility observed in Lin^−^Kit^+^ BM cells corresponded with gene expression changes in CMP, GMP, and MEP populations, which collectively comprise the bulk of Lin^−^Kit^+^ cells. A substantial overlap of genes downregulated in *Arid1a* KO CMP, GMP, and MEP cells and those with closed chromatin in *Arid1a*-deficient Lin^−^Kit^+^ cells was observed (Fig. 6f), suggesting a correlation between closed chromatin and transcriptional inactivation.

### Depletion of ARID1A impairs granulocytic differentiation of NB4 cells

**Fig. 7:**
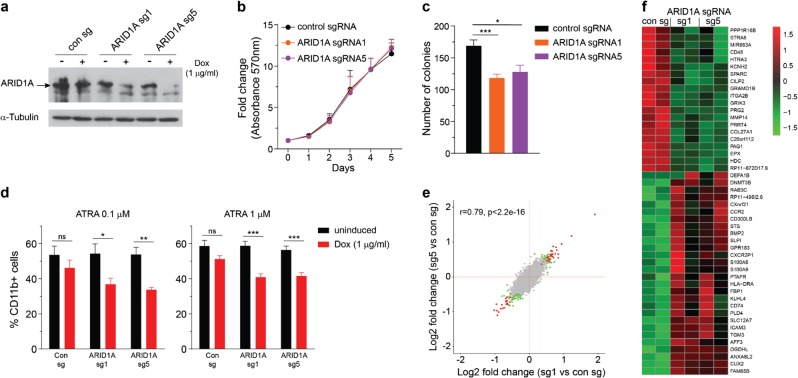
Loss of ARID1A impairs granulocytic differentiation of NB4 cells. **a** Immunoblot shows doxycycline (Dox) induced knockout of ARID1A in NB4 cells expressing Cas9 and sgRNA targeting *ARID1A*. Protein lysate from NB4 cells treated with doxycycline for 7 days were analyzed. **b** Viability of ARID1A-deficient and control NB4 cells was determined using MTT reagent (*n* = 5). **c** Colony-forming ability of *ARID1A* knockout NB4 cells compared with control cells. Cells were plated in media containing methylcellulose, and colonies were enumerated after 9–11 days (*n* = 6). **d** Percentage of CD11b-expressing cells at 48 h after treatment with 0.1 (*n* = 5) and 1 µm ATRA (*n* = 7). **e** Correlation plot compares expression profiles of Dox-treated *ARID1A* sg1 and sg5 NB4 cells compared to Dox-treated control cells. Genes significantly upregulated or downregulated (FDR < 0.1; absolute log_2_ fold change > 0.1) in both sg1- and sg5-expressing NB4 cells are shown in red and those which are significantly altered with only one sgRNA are depicted in green. **f** Heat map shows differential expression of genes significantly altered in both Dox-treated *ARID1A* sg1 and sg5 expressing NB4 cells compared to the Dox-treated control cells (FDR < 0.1). Data are represented as mean ± SEM. *p < 0.05, **p < 0.01, ***p < 0.001, ns = not significant

We have previously reported loss-of-function mutations of *ARID1A* in APL [16]. To investigate the effect of ARID1A deficiency on APL cells, we employed an inducible lentiviral vector system described before [45]. NB4 cells stably expressing Cas9 were transduced with small guide RNA (sgRNA) vector and knockout of *ARID1A* was induced using doxycycline (Dox) (Fig. 7a). Loss of ARID1A did not affect the viability of NB4 cells in short-term liquid culture; but ARID1A-deficient cells had decreased clonogenic potential compared with the control cells (Fig. 7b, c). To assess the effect of ARID1A deficiency on granulocytic differentiation of NB4 cells, we treated control and ARID1A-deficient cells with all-trans retinoic acid (ATRA) for 48 h and examined surface expression of CD11b using flow cytometry. Dox-treated *ARID1A* sgRNA-expressing cells exhibited lower CD11b expression compared to untreated cells and Dox-treated control cells (Fig. 7d). This indicates that *ARID1A* knockout impaired the differentiation potential of NB4 cells. RNA-sequencing revealed that 48 genes were differentially expressed (FDR < 0.1) in both *ARID1A* sg1- and sg5-expressing cells compared to control cells (Fig. 7e, f and Supplementary Table 7). Importantly, overall expression profiles of sg1 and sg5 targeted NB4 cells showed a high correlation (Fig. 7e). GSEA of sg1 and sg5-expressing *ARID1A* deficient NB4 cells showed a high overlap of enriched genesets including those involved in regulation of cell cycle, oxidative phosphorylation and MYC transcriptional network (Supplementary Figure 11a–b). Notably, genesets related to cell cycle (HALLMARK_E2F_TARGETS and HALLMARK_G2M_CHECKPOINT) were enriched in ARID1A-deficient NB4 cells, as well as Arid1a KO *murine* HSCs/precursors, suggesting that ARID1A mediates similar chromatin dynamics and transcriptional regulation in both murine and human cells.

## Discussion

Genetic studies using mouse models indicate that ATP-dependent SWI/SNF chromatin remodeling complex is a crucial regulator of hematopoiesis. While BAF53a, BAF47/Snf5, and BAF45a/PHF10 regulate HSC activity [46–48], other members of SWI/SNF complex play a more lineage-specific role including SMARCD2 in granulopoiesis [49, 50], Brg1 in erythroid development [51] and BAF155 in B cell development [52]. In this study, we have described in depth the role of ARID1A, a principal component of SWI/SNF, in hematopoiesis and examined how its deficiency alters chromatin structure in mouse hematopoietic cells.

In the current study, the function of ARID1A in normal hematopoiesis was elucidated by utilizing two different Cre transgenes, driven by either Vav or Mx1. Vav-iCre mediated deletion of floxed allele commences in fetal HSCs and persists through adult hematopoiesis. Mx1-Cre exerts its effect in mice following stimulation with poly(I:C). Owing to a possible developmental role of ARID1A in fetal/neonatal hematopoiesis, a large majority of ARID1A-deficient mice did not survive at weaning in our Vav-iCre model. Analysis of the hematopoietic compartment revealed largely overlapping phenotypes in both models of ARID1A deficiency, although the consequences of its loss were clearly enhanced in the Vav-iCre model. We believe that this is possibly caused by earlier deletion of *Arid1a* (during embryogenic hematopoiesis) in Vav-iCre model, while in Mx1-Cre model, mice were administered poly(I:C) at 8–14 weeks of age and hematopoietic parameters were analyzed four weeks later. Indeed, an efficient Mx1-Cre mediated deletion of *Arid1a* in BM following poly(I:C) treatment phenocopied largely the Vav-iCre model. Conversely, poly(I:C) administration caused an inefficient deletion of Arid1a in the thymus of Mx1-Cre mice which resulted in less dramatic effect on T cell maturation compared with the Vav-iCre mice.

Loss of ARID1A resulted in accumulation of LTHSCs in the BM, indicating requirement of ARID1A in maintenance of HSC pool size. This is reminiscent of increased HSC frequency observed in fetal liver of constitutive *Arid1a* KO embryos [27]. Results of our in vivo BrdU labeling showed elevated cycling of HSCs, implying decreased quiescence upon loss of ARID1A. Despite their increased number, *Arid1a* KO HSCs possessed poor multilineage reconstitution ability compared with the WT HSCs and were outcompeted in the transplantation settings. Apart from its vital role in maintaining HSC frequency and function, we uncover that ARID1A is also required for normal maturation of both myeloid and lymphoid lineages.

ATAC-seq data demonstrated that chromatin remodeling mediated by ARID1A-containing SWI/SNF complex is critical for hematopoiesis in mice. Deficiency of ARID1A led to a global decrease in open chromatin in myeloid precursors, including at several gene loci, which encode for key regulators of hematopoiesis. Notably, loss of ATAC-seq signal at these loci also correlated with reduced gene expression in myeloid precursors. One of the important downstream targets of ARID1A deficiency is GATA2, which plays an essential role in proliferation and maintenance of hematopoietic stem/progenitor cells, as well as differentiation towards multiple hematopoietic lineages [53]. Another affected gene is *Cd34*, protein encoded by which is expressed on the surface of HSCs and its knockout in mice leads to hematopoietic defects [54]. Similarly, *Cebpa* and *Csf1*, which are crucial regulators of myeloid differentiation [55, 56] also exhibited decreased chromatin accessibility and expression in the ARID1A-deficient cells. Therefore, we postulate that the loss of ARID1A alters chromatin dynamics and ensuing transcriptional changes manifest into impaired myeloid differentiation. Further studies are necessary to explore how loss of ARID1A effects the assembly/composition of SWI/SNF complexes and whether its closely related homolog, ARID1B, plays either an overlapping or distinctive role in chromatin remodeling in hematopoietic cells.

Evidence is emerging that ARID1A is vital for differentiation of various cell types [57–59]. Our study demonstrates that loss of ARID1A does not confer growth advantage to hematopoietic progenitors, but leads to defective differentiation of multiple hematopoietic lineages. For instance, myeloid cells in the BM and spleen of ARID1A-deficient mice displayed reduced surface expression of Gr1, indicating defects in terminal granulopoiesis. The block in myeloid differentiation caused by ARID1A deficiency may have implications in pathogenesis of APL, a subtype of AML in which truncating mutations of *ARID1A* were previously described [16]. In fact, silencing of *ARID1A* in NB4 cells did not affect significantly their growth but impaired the ATRA-induced granulocytic differentiation. Further studies are warranted to investigate whether the impaired differentiation in many hematological disorders including B-cell malignancies and APL is probably in part caused by loss-of-function mutations of *ARID1A*.

Following the discovery of frequent inactivating alterations of *ARID1A*, several approaches to therapeutically target ARID1A-deficient cancer cells have been proposed. These include increased sensitivity of ARID1A-mutated cells to inhibition of either polycomb activity, the PI3K/AKT pathway, the DNA damage checkpoint or the immune checkpoint [60]. Although these studies have focused on synthetic lethal interactions involving inactivation of SWI/SNF activity in solid tumors, particularly in ovarian cancers, which exhibit high mutational frequencies of *ARID1A*, similar strategies can be extended potentially to hematological malignancies harboring mutations of *ARID1A*. For instance, in APL cases with mutation of *ARID1A*, standard therapy (either ATRA or arsenic trioxide) could be combined with an approach directed at ARID1A deficiency, thus leading to potentially improved and personalized therapeutic approaches. Experimental models developed in this study including hematopoietic cell-specific *Arid1a* KO mice and ARID1A-deficient NB4 cells are valuable to explore these therapeutic strategies. For example, mice lacking ARID1A can be crossed with murine models of hematological malignancies for preclinical studies to test precision therapies.

Overall, this study enhances our understanding of the function of SWI/SNF complex in hematopoiesis. It demonstrates that nucleosome remodeling mediated by ARID1A is fundamental for normal hematopoietic development.

## Supplementary information

Supplementary data

Supplementary Table 4

Supplementary Table 5

Supplementary Table 6

Supplementary Table 7
