# Supplementary material for: Chromatin remodeling mediated by ARID1A is indispensable for normal hematopoiesis in mice
Source: Leukemia. 2019 Mar 11;33(9):2291–305. doi: 10.1038/s41375-019-0438-4 (PMC6756219; doi:10.1038/s41375-019-0438-4)
Supplement: Supplementary file 1 — Supplementary data [file 41375_2019_438_MOESM1_ESM.pdf]

Supplementary Information

Chromatin remodeling mediated by ARID1A is indispensable for normal hematopoiesis in mice

Running title: ARID1A is essential for normal hematopoiesis

Lin Han[1,2,6], Vikas Madan[1,6], Anand Mayakonda[1], Pushkar Dakle[1], Teoh Weoi Woon[1], Pavithra Shyamsunder[1], Hazimah Binte Mohd Nordin[1], Zeya Cao[1], Janani Sundaresan[1], Ienglam Lei[3], Zhong Wang[3], and H. Phillip Koeffler[1,4,5]

[1]Cancer Science Institute of Singapore, National University of Singapore, Singapore;

[2]Department of Medicine, Yong Loo Lin School of Medicine, National University of Singapore, Singapore;

[3]Department of Cardiac Surgery, Cardiovascular Research Center, University of Michigan, Ann Arbor, Michigan 48109

[4]Cedars-Sinai Medical Center, Division of Hematology/Oncology, UCLA School of Medicine, Los Angeles, CA, USA;

[5]Department of Hematology-Oncology, National University Cancer Institute of Singapore (NCIS), National University Hospital, Singapore

[6]These authors contributed equally to this work.

Supplementary Materials and Methods

Expression analysis

Different hematopoietic populations were sorted from bone marrow and spleen of C57BL/6 mice. RNA was extracted using RNeasy micro kit (Qiagen) and reverse transcribed using RevertAid First Strand cDNA Synthesis Kit (Thermo Scientific). Transcript levels of *Arid1a* were determined using SYBR fast qPCR Master Mix (Kapa Biosystems).

Analysis of Cre-recombinase mediated deletion of *Arid1a*

Efficiency of Cre-recombinase mediated excision of *Arid1a* exon 9 was determined in peripheral blood leukocytes, two weeks after poly(I:C) injection. PCR analysis was performed using primers GTAATGGGAAAGCGACTACTGGAG and TGTTCATTTTTGTGGCGGGAG and PCR products corresponding to the floxed allele (845 bp) and the deleted allele (298 bp) were resolved on agarose gel. Mice were analyzed 4-5 weeks after the last injection of poly(I:C) and deletion of floxed allele in BM cells and lymphoid organs was verified using PCR as well as immunoblotting.

Colony re-plating assay

Bone marrow cells were plated in methylcellulose media supplemented with murine stem cell factor (SCF), interleukin 3 (IL-3) and human interleukin 6 (IL-6) and erythropoietin (MethoCult GF M3434; StemCell Technologies). Colonies were enumerated after 9-10 days and the cells were harvested and counted for re-plating.

In vitro deletion of ARID1A

MSCV-IRES-Puro (MIP) retroviral vector expressing Cre recombinase was used to excise the floxed allele in vitro. To generate viral particles, Plat-E cells were transfected with either empty vector (EV) or Cre-expressing plasmid using jetPRIME transfection reagent (Polyplus-transfection). At 48h and 72h post transfection, virus supernatants were collected and concentrated using Amicon Ultra-15 Centrifugal Filter (Merck Millipore). $Lin^{-}Kit^{+}$ BM cells from 129 x C57BL/6 mix background *Arid1a*$^{f/f}$ mice were sorted and maintained in IMDM containing 15% FBS and supplemented with recombinant interleukin-3, interleukin-6 and stem cell factor. Cells were transduced twice, 24h apart, using RetroNectin reagent (Takara) and selected with 2 µg/ml puromycin for one week. Genomic DNA was extracted from puromycin-selected cells and efficiency of deletion was determined using PCR. For clonogenic assays, 5,000 cells were plated in 35 mm dishes, and colonies were counted after 8 days.

Peripheral blood analysis

Complete peripheral blood counts were analysed using Abbott Cell-Dyn 3700 Hematology Analyzer (Abbott Laboratories).

BrdU incorporation assay

Three weeks after the last poly(I:C) injection, *Arid1a*$^{f/f}$*;Mx1-Cre*$^{+}$ and *Arid1a*$^{f/f}$*;Mx1-Cre*$^{-}$ mice received bromodeoxyuridine (BrdU) in drinking water (1 mg/ml) for 1 week. Bone marrow cells were harvested and Lin^{-} cells were enriched using Dynabeads Sheep

anti-rat IgG. Following staining with fluorochrome-conjuagted antibodies, cells were fixed and permeabilized using BrdU Flow Kit (BD Biosciences) as per the manufacturer's instruction. Cells were then stained with anti-BrdU antibody and Hoechst 33342 dye (1 µg/ml) and acquired on LSRII flow cytometer (BD Biosciences).

Non-competitive reconstitution assays

Two million BM cells from either *Arid1a*$^{f/f}$*;Mx1-Cre*$^{+}$ or *Arid1a*$^{f/f}$*;Mx1-Cre*$^{-}$ mice were injected intravenously into lethally irradiated B6.SJL recipients. Following successful engraftment at four weeks post transplantation, *Arid1a* deletion was induced using poly(I:C). At 4, 8, 12 and 16 weeks post poly(I:C) injection, peripheral blood was collected and differential leukocyte counts were analysed.

CRISPR knockout of ARID1A in NB4 cells

A dual lentiviral vector system previously described[1] was used for inducible depletion of ARID1A in NB4 cells (provided by Dr Wee Joo Chng, Singapore). sgRNA sequences were cloned into BsmbI site of FgH1tUTG lentiviral vector (Addgene # #70183). The target sequence for human *ARID1A* sgRNA1 and sgRNA5 are GAAAGCGAGGGCCCCGCCGT and GCTTCGGGCAACCCTACGGC, respectively. As a control, sgRNA targeting lacZ gene of *Escherichia coli* (TGCGAATACGCCCACGCGAT) was used. NB4 cells were initially transduced with FUCas9Cherry (Addgene #70182), and mCherry^{+} cells were sorted and transduced with FgH1tUTG vector expressing either *ARID1A* sgRNA or control sgRNA along with a GFP reporter gene. Double positive (mCherry^{+}GFP^{+}) cells were sorted and

maintained in medium containing tetracycline-free FBS. To induce *ARID1A* knockout, cells were cultured in media containing 1 μg/ml doxycycline for 7 days.

Assays of cellular proliferation and differentiation of NB4 cells

Seven days after incubation with doxycycline to induce ARID1A depletion, viability, colony and differentiation assays were performed as described previously[2] in doxycycline-free media. For in vitro differentiation, cells were incubated with ATRA (1 μM and 0.1 μM) for 48 hours and assessed using flow cytometry for CD11b expression. For RNA isolation, following doxycycline treatment, cells were cultured further in doxycycline-free media for 2 days and RNA was extracted using RNeasy Mini Kit (Qiagen).

Analysis of RNA-sequencing data

Transcript level fragment counts were summarized to gene level using TxImport Bioconductor package, and differential analysis was performed using DESeq2 v1.18.1[3,4]. Gene expression was quantified in FPKM units using DESeq2 fpkm command and was used for all downstream analysis and plotting. All other test-statistics and plotting were performed using R 3.4.0. For GSEA (v2.2.2), we used all "active transcripts" with mean expression of 0.5 FPKM to identify significantly enriched gene sets among MSigDB Hallmark gene sets[5]. For CMP, GMP and MEP cells, the Signal2Noise metric was used for ranking the genes. For NB4 and LT-HSC cells, GseaPreranked analysis was done with the genes ranked by the log fold changes.

ATAC-seq library preparation

50,000 sorted cells were washed with cold PBS and suspended in lysis buffer (10mM Tris.Cl, pH 7.4, 10 mM NaCl, 3 mM $MgCl_2$, 0.1% (v/v) Igepal CA-630) for 10-15 min on ice. Cells were centrifuged and supernatant was discarded. Nuclei pellet was resuspended in transposition reaction mix (25 µl Nextera 2X reaction buffer, 2.5 µl Nextera Tn5 Transposase and 22.5 µl nuclease-free water). After transposition reaction at 37°C for 30 min, DNA was purified using MinElute PCR Purification Kit (Qiagen). Transposed DNA was amplified (72°C for 5 min; 98°C for 30 sec; 13 cycles of 98°C for 10 sec, 63°C for 30 sec, and 72°C for 1 min) using NEB Next High-Fidelity 2X PCR Master Mix. Libraries were eluted using Qiagen MinElute PCR Purification Kit and purified on PAGE gel (NovexTM TBE Gels, 4-20%; EC6225BOX), followed by staining with SYBR Safe DNA Gel Stain (Invitrogen; S33102). Transposed DNA libraries were sequenced on HiSeq 4000.

ChIP-sequencing

FACS sorted Lin^- BM cells or 32D cells were incubated with 1% formaldehyde for 10 min to crosslink DNA-protein complexes. Following quenching with 0.2 M glycine for 5 min, chromatin was sonicated in Lysis buffer (1% SDS, 50 mM Tris-HCl, 5mM EDTA) using EpiShear Probe Sonicator (Active Motif) (14 rounds of 30 sec on/off cycle). Sheared chromatin was incubated with Dynabeads Protein A + Dynabeads Protein G mixture (1:1), previously conjugated with antibodies (H3K4me3: Merck Millipore, 04-745); H3K27ac: Abcam, ab4729; ARID1A: Abcam, ab182560 and GeneTex, GTX129433; Rabbit IgG isotype control, ab171870) for overnight at 4°C. Beads were then washed; chromatin was eluted in 1% SDS, 0.1M sodium bicarbonate and

reverse-crosslinked at 65°C for 12 hours. Immunoprecipitated DNA was extracted using QIAquick PCR Purification Kit (Qiagen) and DNA quality was assessed on 2100 Bioanalyzer, prior to library preparation. Following PCR amplification and size selection (100-300 bp), libraries were sequenced on HiSeq4000. For ChIP-PCR analysis, immunoprecipitated DNA and input DNA were amplified using SYBR fast qPCR Master Mix (Kapa Biosystems). Primers used for ChIP-PCR are listed in Supplementary Table 2.

For analysis of sequencing data, 50 bp single-end reads were aligned to the mm10 reference genome using the bowtie v1.2.2 aligner[6]. PCR duplicates were marked using picard tools (http://broadinstitute.github.io/picard). Peaks were identified using MACS2 v2.1.1.20160309 with FDR cut-off of 0.01[7], while simultaneously generating bedgraph files in Reads Per Million scale. Input signal was subtracted from ChIP-seq signal and bedgraph files were converted to binary bigwig files. Heatmaps around Refseq transcripts and peak centers were drawn using deeptools[8]. Peaks were annotated using homer annotatePeaks.pl script[9].

Supplementary References

1. Aubrey BJ, Kelly GL, Kueh AJ, et al. An inducible lentiviral guide RNA platform enables the identification of tumor-essential genes and tumor-promoting mutations in vivo. *Cell Rep*. 2015;10(8):1422-1432.
2. Madan V, Shyamsunder P, Han L, et al. Comprehensive mutational analysis of primary and relapse acute promyelocytic leukemia. *Leukemia*. 2016;30(8):1672-1681.
3. Soneson C, Love MI, Robinson MD. Differential analyses for RNA-seq: transcript-level estimates improve gene-level inferences. *F1000Res*. 2015;4:1521.
4. Love MI, Huber W, Anders S. Moderated estimation of fold change and dispersion for RNA-seq data with DESeq2. *Genome Biol*. 2014;15(12):550.
5. Subramanian A, Tamayo P, Mootha VK, et al. Gene set enrichment analysis: a knowledge-based approach for interpreting genome-wide expression profiles. *Proc Natl Acad Sci U S A*. 2005;102(43):15545-15550.
6. Langmead B, Trapnell C, Pop M, Salzberg SL. Ultrafast and memory-efficient alignment of short DNA sequences to the human genome. *Genome Biol*. 2009;10(3):R25.

7. Zhang Y, Liu T, Meyer CA, et al. Model-based analysis of ChIP-Seq (MACS). *Genome Biol*. 2008;9(9):R137.
8. Ramirez F, Dundar F, Diehl S, Gruning BA, Manke T. deepTools: a flexible platform for exploring deep-sequencing data. *Nucleic Acids Res*. 2014;42(Web Server issue):W187-191.
9. Heinz S, Benner C, Spann N, et al. Simple combinations of lineage-determining transcription factors prime cis-regulatory elements required for macrophage and B cell identities. *Mol Cell*. 2010;38(4):576-589.

Supplementary Figure 1

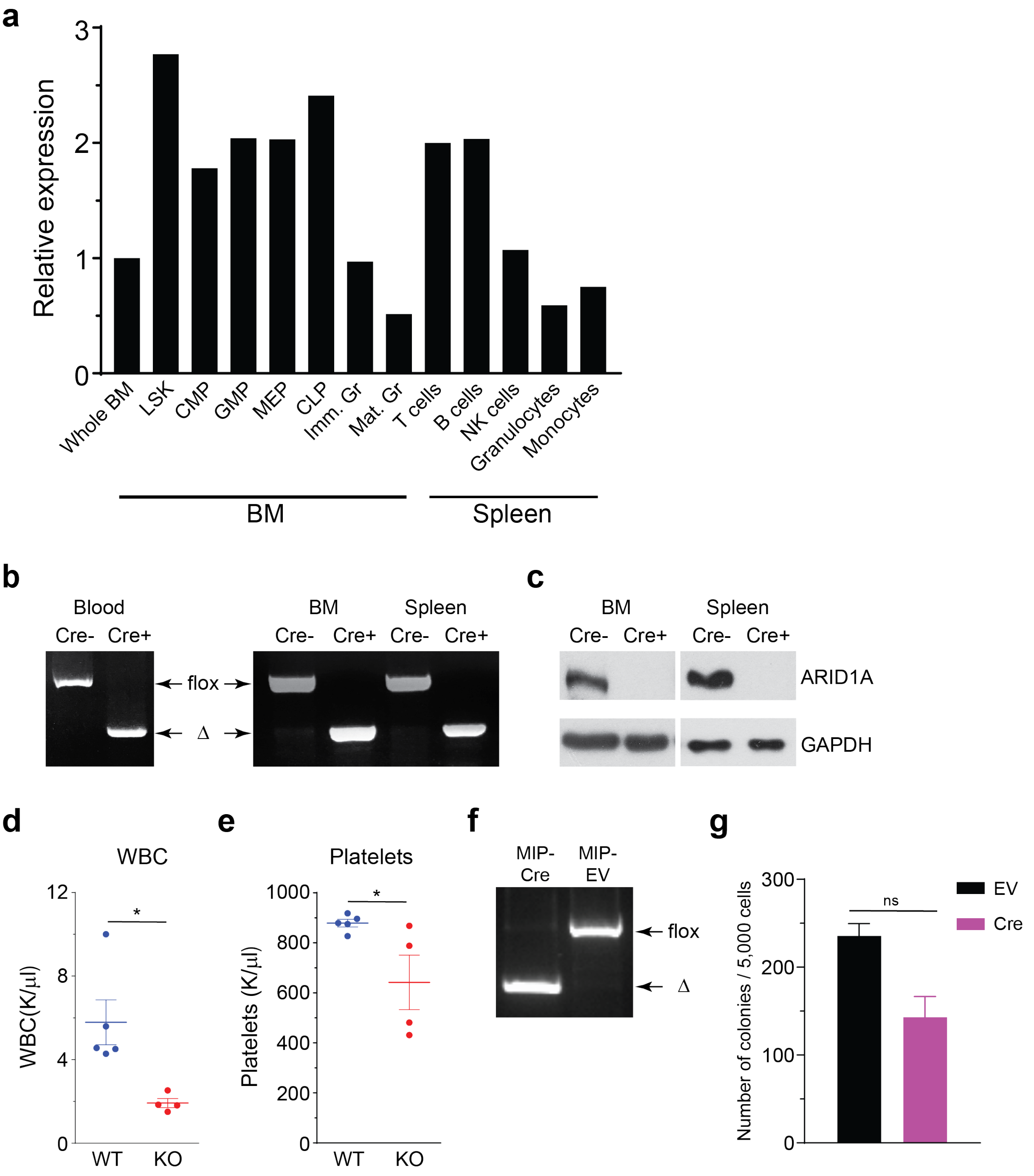

Supplementary Figure 1 (**a**) Transcript levels of *Arid1a* (RT-PCR) in purified populations sorted from bone marrow (BM) and spleen of C57BL/6 mice. *β-actin* was used as endogenous control. (**b**) PCR analysis for floxed and deleted (Δ) *Arid1a* alleles using genomic DNA from blood, BM and spleen cells from *Arid1a*$^{f/f}$*;Vav-iCre*$^{+}$ (Cre+) and control mice (Cre-) as template. (**c**) Western blot analysis for ARID1A expression in BM and spleen of *Arid1a*$^{f/f}$*;Vav-iCre*$^{+}$ and control mice. (**d-e**) WBC (d) and platelet (e) counts in peripheral blood of *Arid1a*$^{f/f}$*;Vav-iCre*$^{+}$ and control mice aged 6-17 weeks. (**f**) PCR analysis for deletion of *Arid1a* exon 9 in Lin^{-}Kit^{+} cells from *Arid1a*$^{f/f}$ mice

transduced ex vivo with either MSCV-IRES-Puro-Cre (MIP-Cre) or empty vector (MIP-EV). (**g**) Number of colonies obtained from $Lin^{-}Kit^{+}$ cells (from $Arid1a^{f/f}$ mice) expressing either Cre recombinase or empty vector (n=2). Data represent mean ± SEM. *p <0.05, ns = not significant.

Supplementary Figure 2

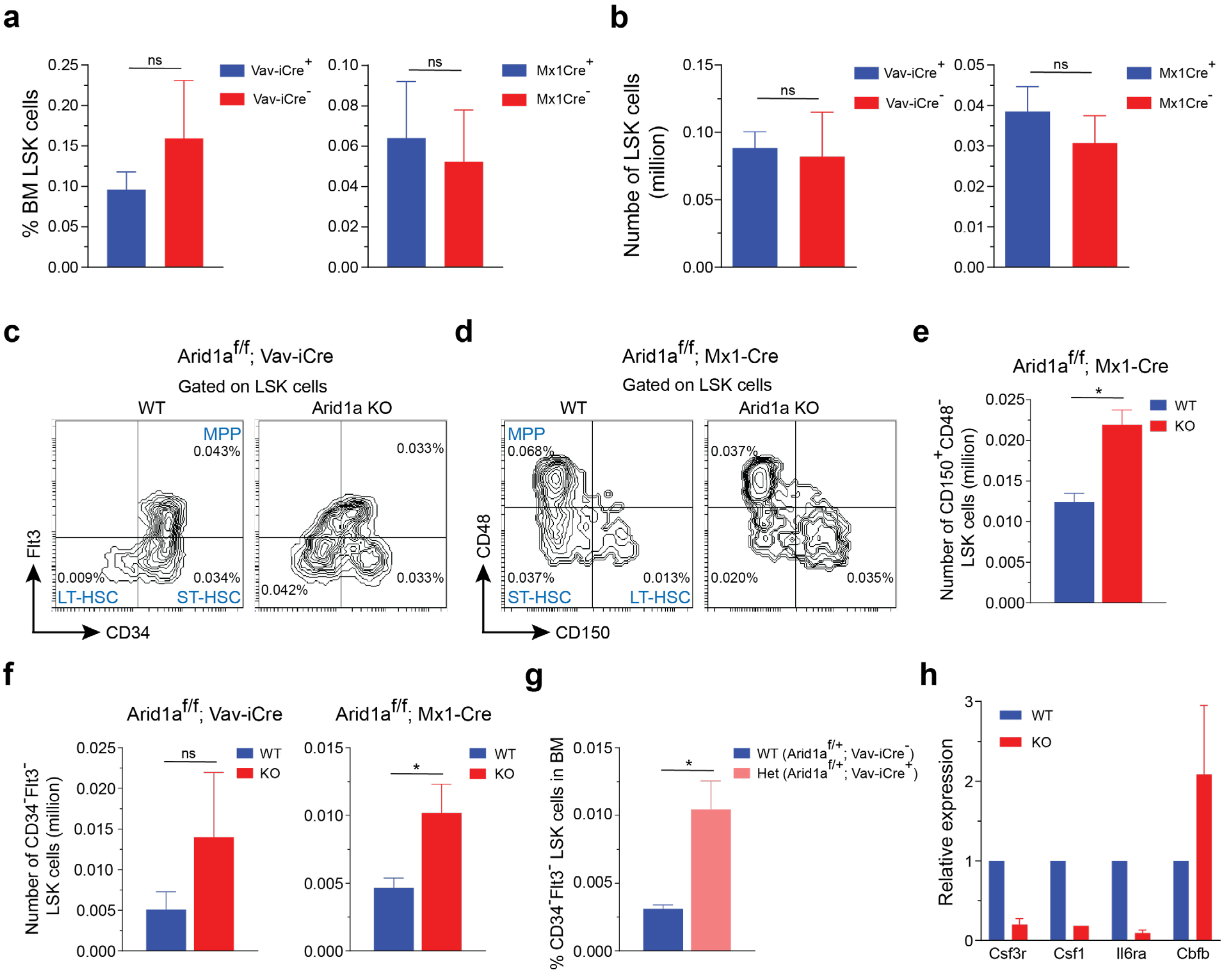

Supplementary Figure 2 (**a-b**) Proportion (a) and absolute number (b) of BM LSK cells in the WT and *Arid1a* KO mice in both Vav-iCre (n=5) and Mx1-Cre (n=6) models. (**c**) Representative staining for HSCs in the BM of *Arid1a*$^{f/f}$*;Vav-iCre*$^{+}$ and control mice. LSK cells were categorised as LTHSCs (CD34^{-}Flt3^{-} LSK), STHSCs (CD34^{+}Flt3^{-} LSK) and MPPs (CD34^{+}Flt3^{+} LSK) using CD34 and Flt3 antibodies. (**d**) Representative plots show staining of HSCs based on SLAM markers, CD150 and CD48, in *ARID1A*$^{f/f}$*;Mx1-Cre*$^{+}$ and *ARID1A*$^{f/f}$*;Mx1-Cre*$^{-}$ mice, four weeks after poly(**I**:C) treatment. (**e**) Number of LTHSCs (CD150^{+}CD48^{-} LSK cells) in BM of *Arid1a*$^{f/f}$*;Mx1-Cre*$^{+}$ and *Arid1a*$^{f/f}$*;Mx1-Cre*$^{-}$ mice, four weeks after poly(I:C) injection (n=3). (**f**) Number of LTHSCs (CD34^{-}Flt3^{-} LSK) in the bone marrow of WT and KO mice, in Vav-iCre (n=3) and Mx1-Cre (n=6) models. (**g**) Percentages of CD34^{-}Flt3^{-} LSK cells in the bone marrow of WT and *Arid1a* heterozygous (Het) mice (n=4). (**h**) Quantitative PCR analysis for transcript levels of *Csf3r*, *Csf1*, *IL6ra* and *Cbfb* in LT-HSCs sorted from BM of *Arid1a*$^{f/f}$*;Mx1-Cre*$^{+}$ and *Arid1a*$^{f/f}$*;Mx1-Cre*$^{-}$ mice, 4 weeks after poly(I:C) injection. *β-actin* were used as endogenous control. Data represent mean ± SEM. *p <0.05, ns = not significant.

Supplementary Figure 3

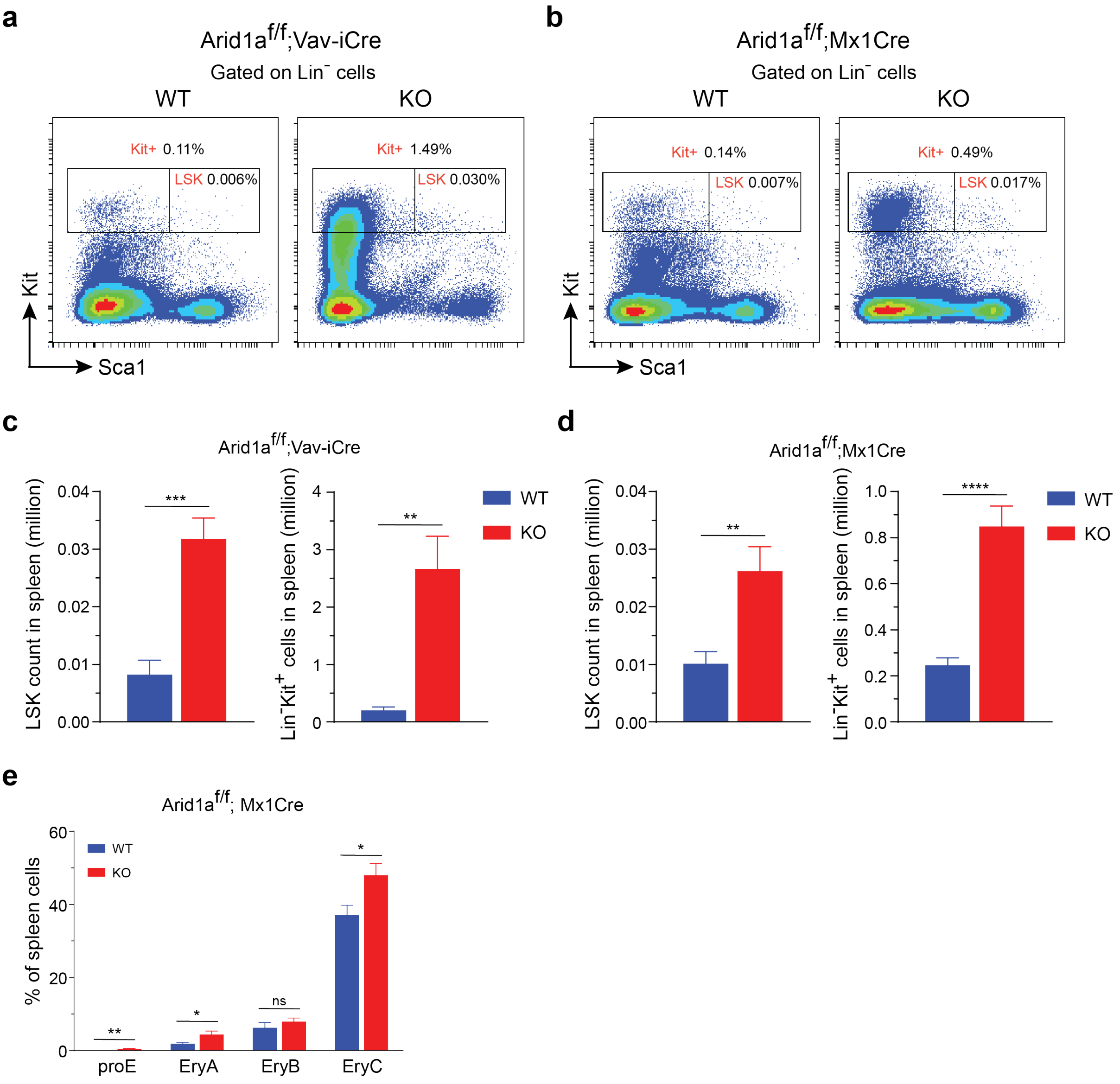

Supplementary Figure 3 (**a-b**) Representative flow cytometry plots show staining of Lin$^-$Kit$^+$ and LSK cells in the spleens of *Arid1a* KO and WT mice, in both Vav-iCre (a) and Mx1-Cre (b) models. (**c-d**) Absolute numbers of LSK and Lin$^-$Kit$^+$ cells in the spleen of Vav-iCre (c) (n=5) and Mx1-Cre (d) (n=9) mice. (**e**) Proportion of erythroid progenitors in the spleen of *Arid1a$^{f/f}$;Mx1-Cre$^+$* and *Arid1a$^{f/f}$;Mx1-Cre$^-$* mice, four weeks after poly(I:C) treatment (n=9). Error bars represent SEM. *p <0.05, **p<0.01, ***p<0.001, ****p<0.0001, ns = not significant.

Supplementary Figure 4

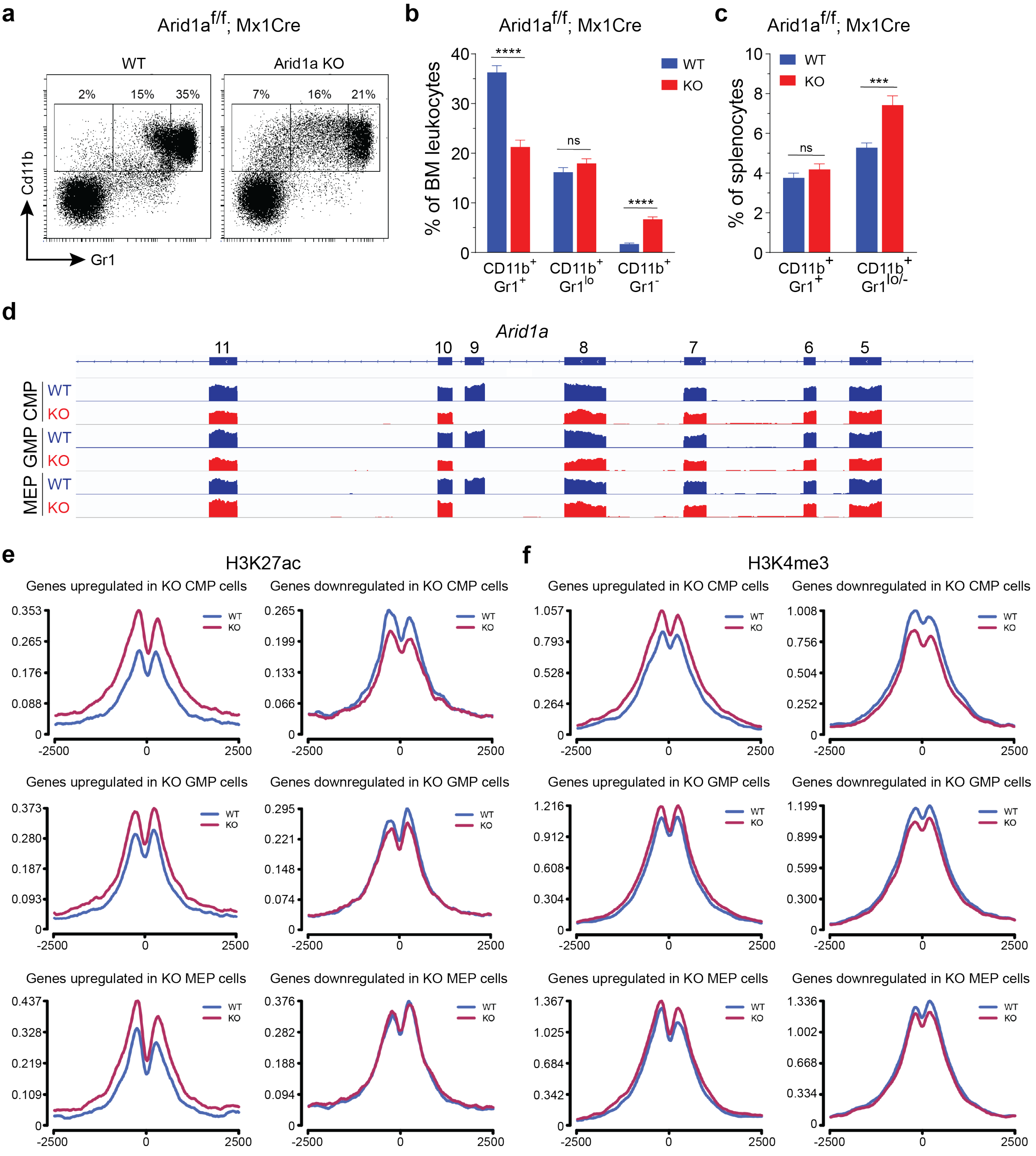

Supplementary Figure 4 (**a**) Representative flow cytometric analysis of CD11b and Gr1 expression in BM of WT and *Arid1a* KO (Mx1-Cre) mice. (**b**) Proportion of CD11b$^+$Gr1$^+$, CD11b$^+$Gr1^{lo} and CD11b$^+$Gr1$^-$ myeloid cells in the BM of *Arid1a$^{f/f}$;Mx1-Cre$^+$* and *Arid1a$^{f/f}$;Mx1-Cre$^-$* mice four weeks after poly(I:C) injection (n=6). (**c**) Proportion of myeloid cells in the spleen of *Arid1a$^{f/f}$;Mx1-Cre$^+$* and *Arid1a$^{f/f}$;Mx1-Cre$^-$* mice four weeks after administration of poly(I:C) (n=9). (**d**) IGV snapshot of *Arid1a* locus shows efficient deletion of exon 9 in CMP, GMP and MEP cells sorted from poly(I:C) injected *Arid1a$^{f/f}$;Mx1-Cre$^+$* mice. (**e-f**) Intensity of H3K27ac (e) and H3K4me3

(f) ChIP-seq signals in WT and *Arid1a* KO Lin^- BM cells for genes differentially expressed in CMP, GMP and MEP populations. Data are represented as mean ± SEM. ***p<0.001, ****p<0.0001, ns = not significant.

Supplementary Figure 5

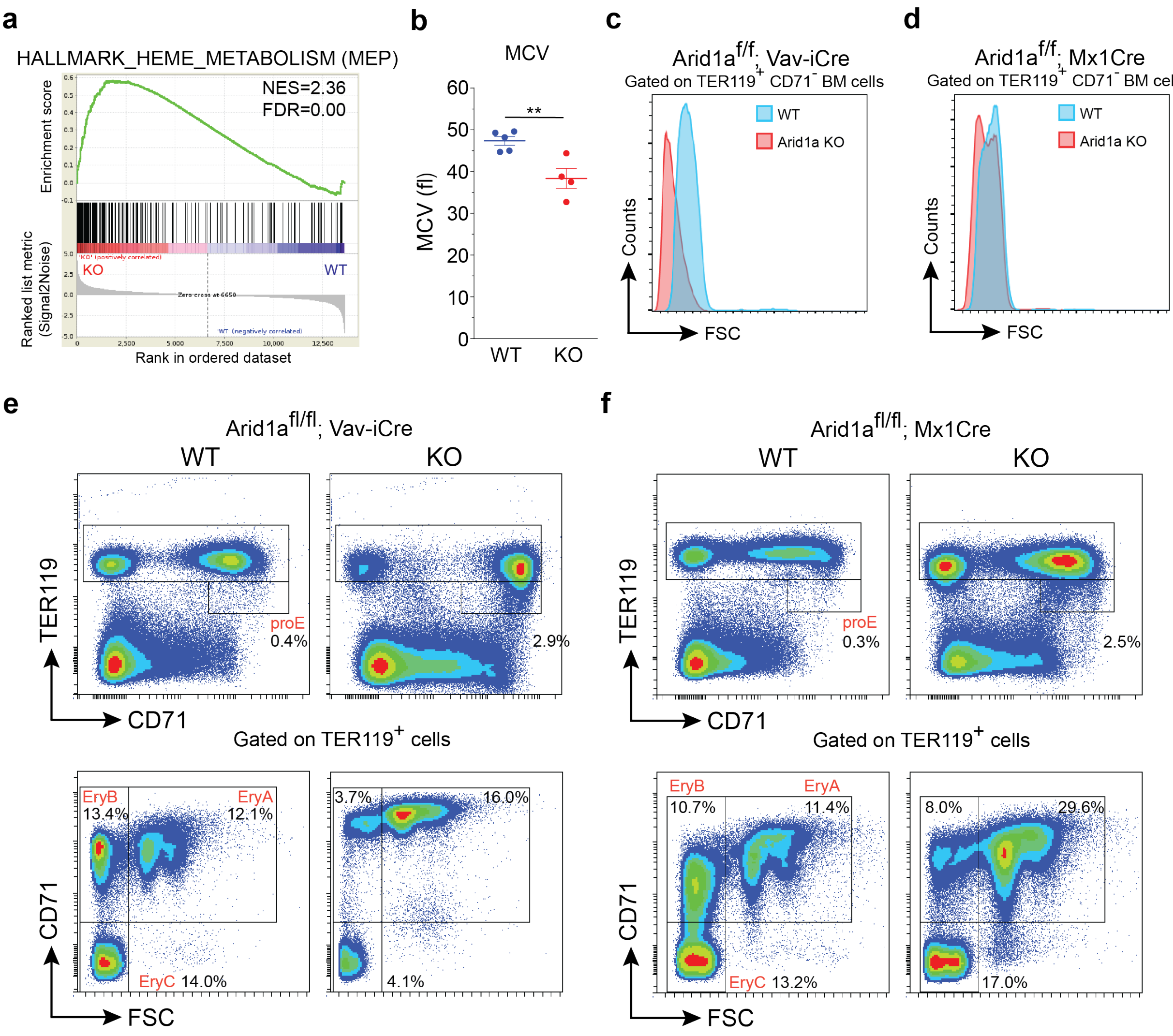

Supplementary Figure 5 (**a**) GSEA plot for HALLMARK_HEME_METABOLISM in the comparison of WT and *Arid1a* KO (Mx1-Cre) MEP cells. NES: Normalized enrichment score. (**b**) Comparison of red blood cell volume (MCV) in peripheral blood of *Arid1a*$^{f/f}$*;Vav-iCre*$^{+}$ and control mice. (**c-d)** Histogram overlay of forward scatter (FSC) for mature erythrocytes (TER119^{+}CD71^{-}) in the BM of WT and *Arid1a* deficient mice, in both Vav-iCre (c) and Mx1-Cre (d) models. (**e-f**) Representative FACS staining for erythroid development in BM of Vav-iCre (e) and Mx1-Cre (f) models of *Arid1a* deficiency. Cells were stained with CD71 and TER119 antibodies and gated into four successive stages of erythrocyte development (proE: CD71^{+}TER119^{lo}, EryA: CD71^{+}TER119^{+}FSC^{hi}, EryB: CD71^{+}TER119^{+}FSC^{lo} and EryC: CD71^{-}TER119^{+}FSC^{lo}). Error bars represent SEM. **p<0.01.

Supplementary Figure 6

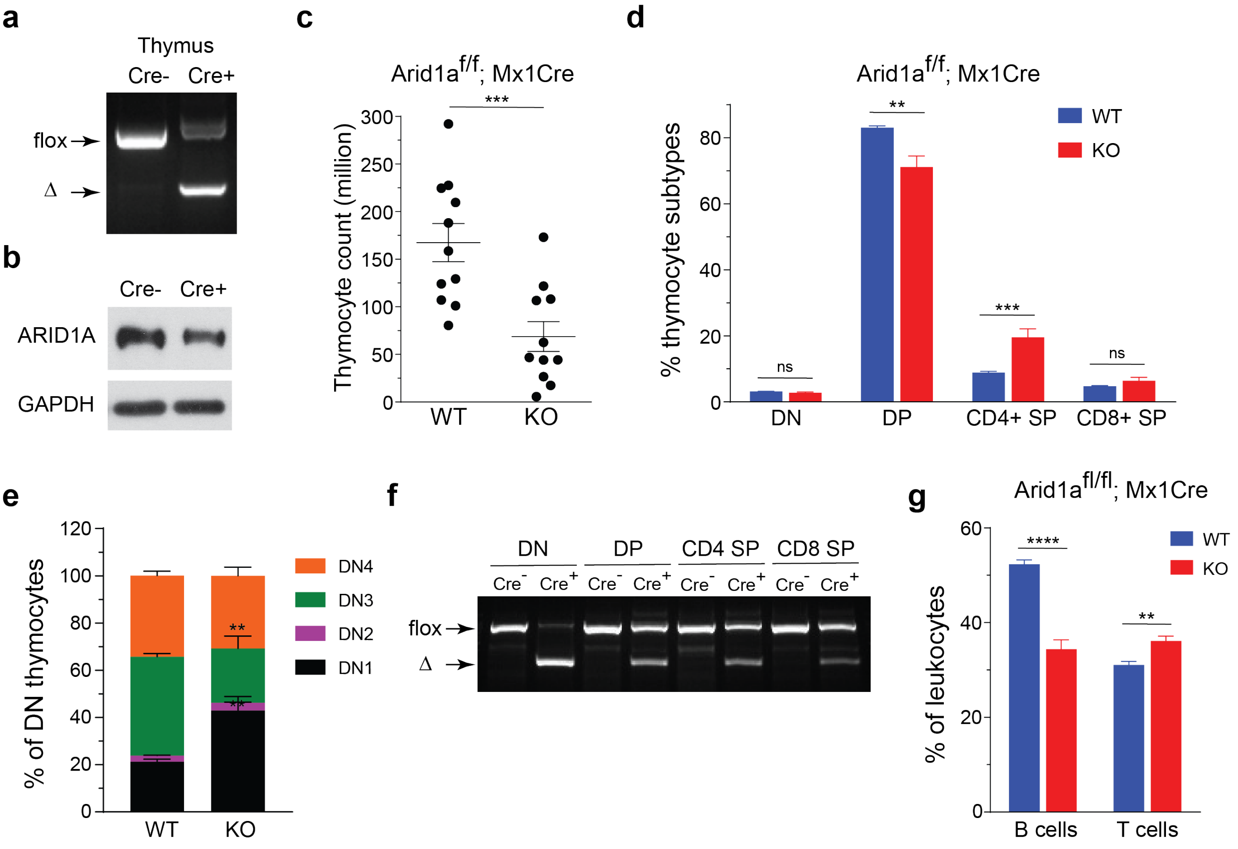

Supplementary Figure 6 (**a**) Representative PCR image for floxed and deleted *Arid1a* alleles in the thymocytes of *Arid1a$^{f/f}$;Mx1-Cre^{+}* and *Arid1a$^{f/f}$;Mx1-Cre^{-}* mice, four weeks after poly(I:C) injection. (**b**) ARID1A protein levels determined four weeks after poly(I:C) injection in the thymus of *Arid1a$^{f/f}$;Mx1-Cre^{+}* and *Arid1a$^{f/f}$;Mx1-Cre^{-}* mice. (**c**) Number of thymocytes in *Arid1a$^{f/f}$;Mx1-Cre^{+}* (KO) and *Arid1a$^{f/f}$;Mx1-Cre^{-}* (WT) mice four weeks after poly(I:C) injection. (**d**) Percentages of DN, DP, CD4^{+} and CD8^{+} SP cells in the thymi of *Arid1a$^{f/f}$;Mx1-Cre^{+}* and *Arid1a$^{f/f}$;Mx1-Cre^{-}* mice four weeks after poly(I:C) injection (n=9). (**e**) Proportion of DN1, DN2, DN3 and DN4 cells within the DN compartment in control and ARID1A-deficient mice in Mx1-Cre deletion model (n=9). (**f**) PCR analysis shows Mx1-Cre-mediated deletion of *Arid1a* allele in sorted DN, DP, CD4^{+} and CD8^{+} SP thymocytes following administration of poly(I:C). (**g**) Percentages of B and T cells in the spleens of *Arid1a$^{f/f}$;Mx1-Cre^{+}* and *Arid1a$^{f/f}$;Mx1-Cre^{-}* mice four weeks after poly(I:C) injection (n=8). Data represent mean ± SEM. **p<0.01, ***p<0.001, ****p<0.0001, ns = not significant.

Supplementary Figure 7

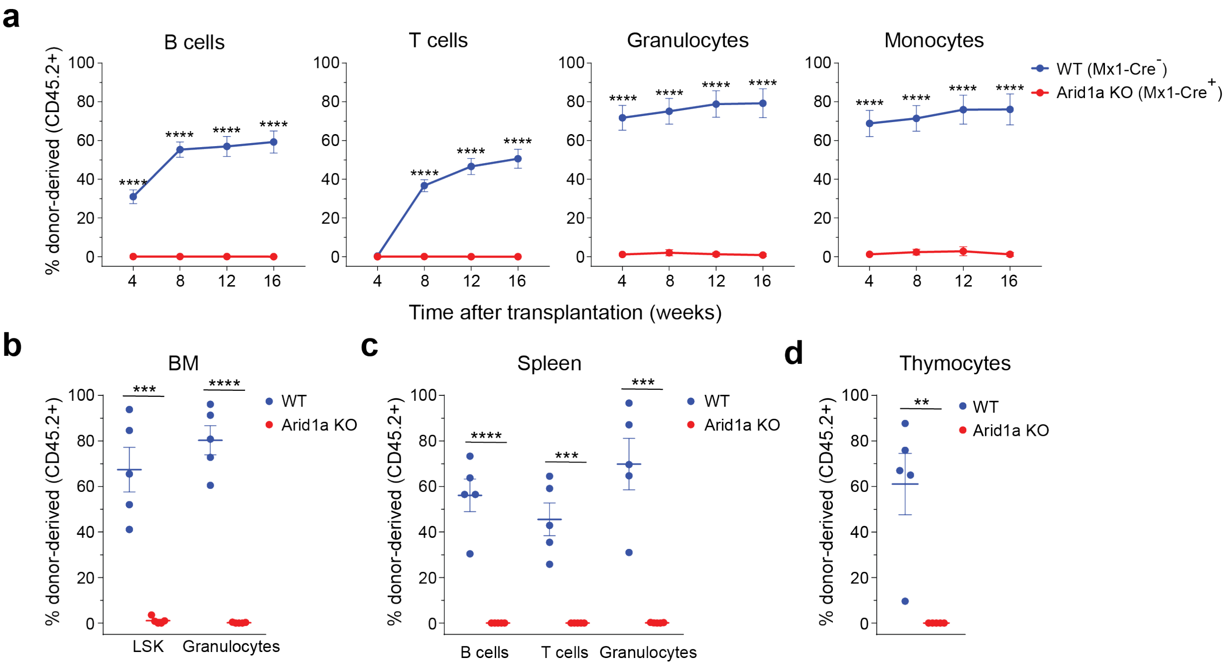

Supplementary Figure 7 (**a**) Proportion of donor-derived lymphoid and myeloid cells in the peripheral blood of mice transplanted with either WT or *Arid1a* KO LTHSCs in competitive repopulation assay. Reconstitution was analysed every four weeks after transplantation. (**b-d**) Donor chimerism in the BM (b), spleen (c), and thymus (d) of mice transplanted as in (a). Mice were analysed 16-25 weeks after transplantation. Error bars represent SEM. **p<0.01, ***p<0.001, ****p<0.0001, ns = not significant.

Supplementary Figure 8

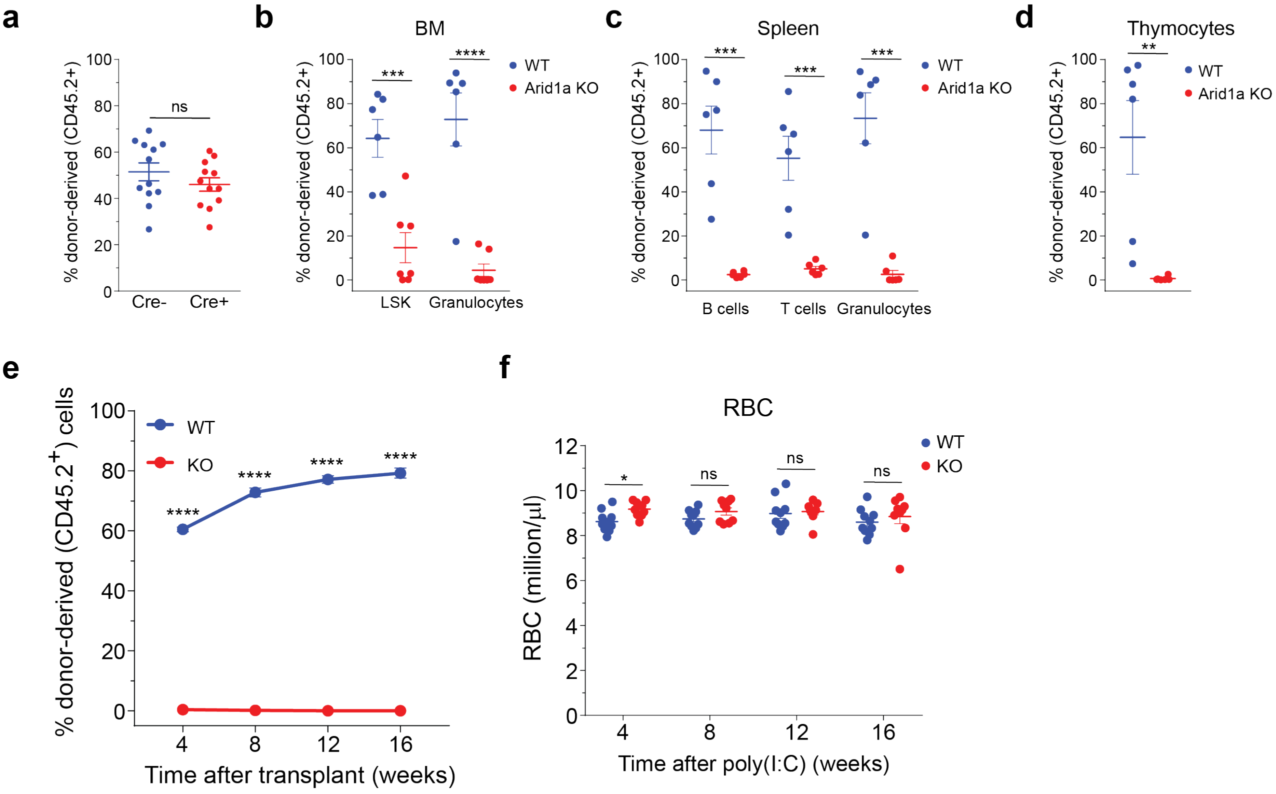

Supplementary Figure 8 (**a**) Proportion of donor-derived cells in recipient mice transplanted with BM cells (either *Arid1a*$^{f/f}$*;Mx1-Cre*$^{+}$ or *Arid1a*$^{f/f}$*;Mx1-Cre*$^{-}$ BM mixed with competitor BM (1:1)) at four weeks post transplantation (prior to administration of poly(I:C)). (**b-d**) Donor chimerism in the BM (b), spleen (c), and thymus (d) of recipient mice transplanted as in (a). Mice were analysed at 30-50 weeks after poly(I:C) injection. (**e**) Average donor contribution (CD45.2^{+}) to peripheral blood leukocytes in mice transplanted with either *Arid1a*$^{f/f}$*;Vav-iCre* or control BM cells in competitive repopulation assay (6 recipients/genotype). Reconstitution was assessed in peripheral blood every four weeks post transplantation. (**f**) RBC counts in peripheral blood of recipients transplanted with either *Arid1a*$^{f/f}$*;Mx1-Cre*$^{+}$ or *Arid1a*$^{f/f}$*;Mx1-Cre*$^{-}$ BM in non-competitive repopulation assay. Blood was drawn every 4 weeks post poly(I:C) injection (9 recipients for *ARID1A*$^{f/f}$*;Mx-Cre*$^{+}$, 10 recipients for *Arid1a*$^{f/f}$*;Mx1-Cre*$^{-}$). Data represent mean ± SEM. *p <0.05, **p<0.01, ***p<0.001, ****p<0.0001, ns = not significant.

Supplementary Figure 9

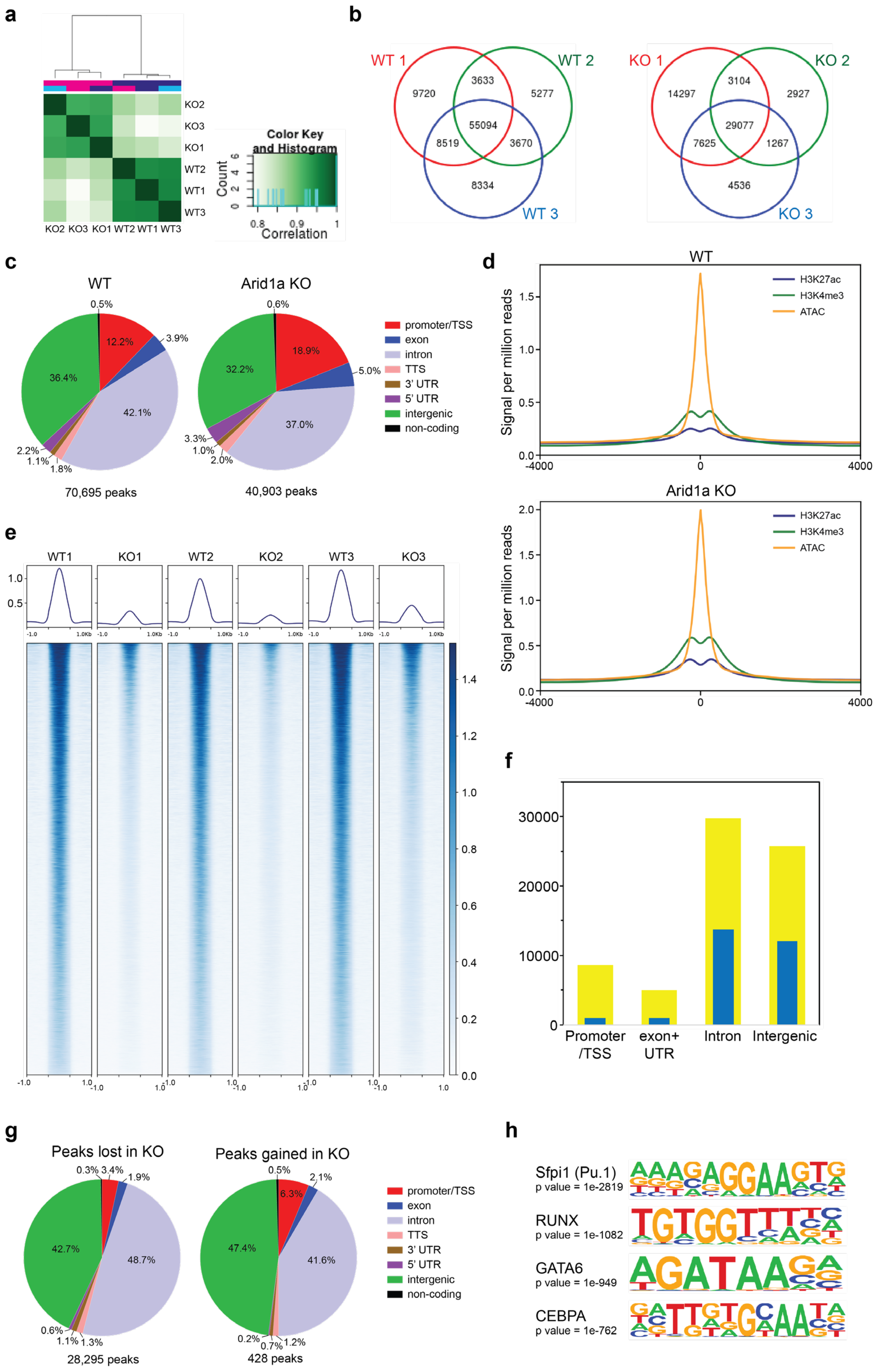

Supplementary Figure 9 (**a**) Heat map generated using DiffBind package shows correlation between each WT and *Arid1a* KO sample based on ATAC-seq read counts. (**b**) Venn diagrams show number of ATAC-seq peaks for each replicate. (**c**) Pie charts show the genomic distribution of ATAC-seq peaks (detected in at least two of the three replicates) in WT and *Arid1a* KO $Lin^{-}Kit^{+}$ BM cells. Cells were sorted from *Arid1a*$^{f/f}$*;Mx1-Cre*$^{+}$ and *Arid1a*$^{f/f}$*;Mx1-Cre*$^{-}$ mice four weeks after administration of poly(**I**:C). (**d**) Signal density for H3K4me3, H3K27ac and ATAC-seq in WT and *Arid1a* KO cells (centered at the summit of ATAC-seq peaks). (**e**) Heat maps show ATAC-seq peaks (scaled to 1 Kb) significantly closed in *Arid1a* KO cells compared with WT cells in three replicates. (**f**) Number of ATAC-seq peaks significantly closed in KO cells (blue) relative to the total number of peaks (yellow) in WT cells at annotated genomic elements. (**g**) Distribution of ATAC-seq peaks either lost or enriched in KO $Lin^{-}Kit^{+}$ BM cells. TSS: transcriptional start site, TTS: transcriptional termination site. (**h**) Top four motifs (Homer de novo motif analysis) enriched in loci with significantly reduced ATAC-seq signal in *Arid1a* KO cells.

Supplementary Figure 10

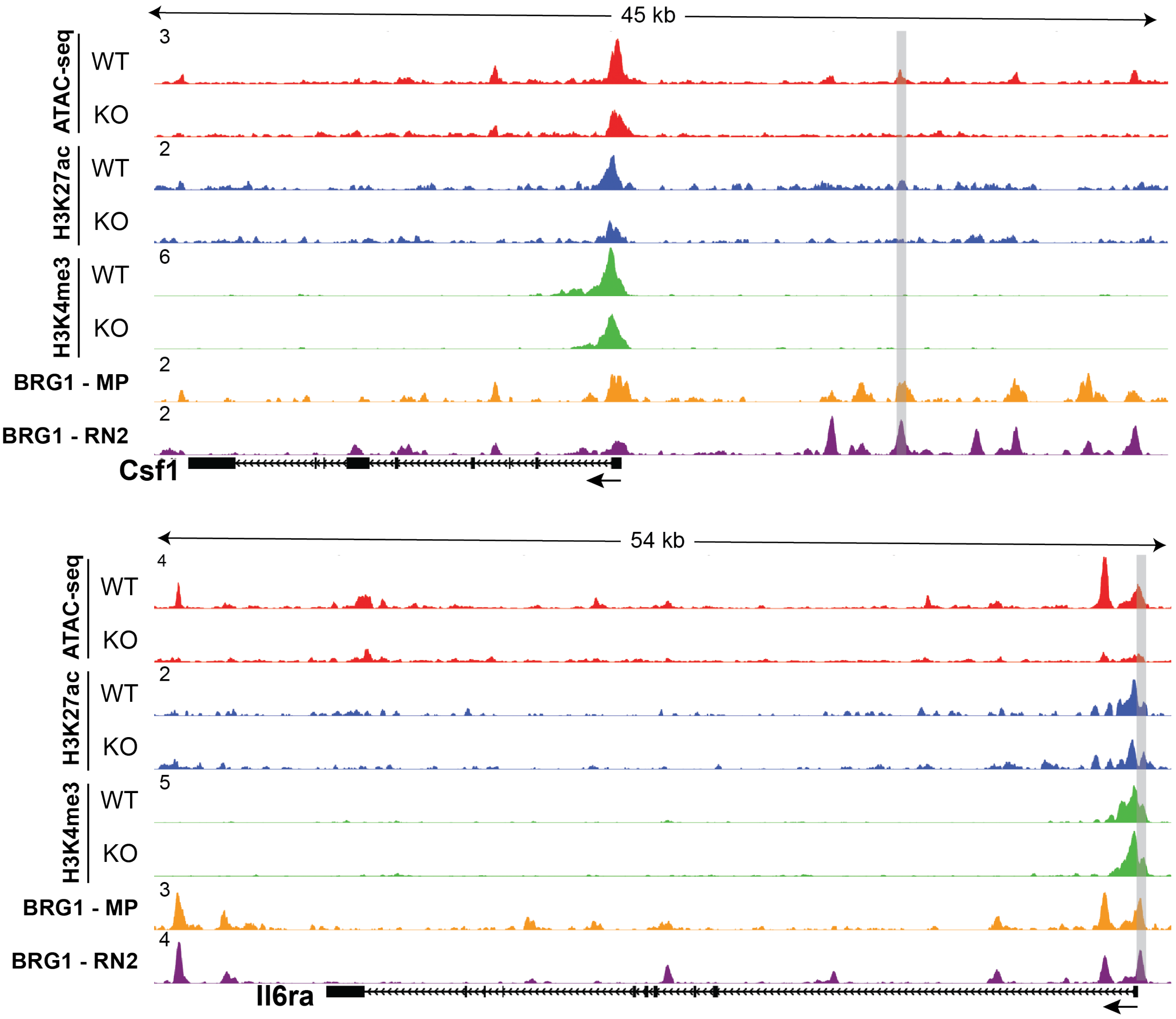

Supplementary Figure 10. Representative tracks for ATAC-seq signal (Lin$^-$Kit$^+$ BM cells), H3K27ac and H3K4me3 marks (both Lin$^-$ BM cells) and BRG1 ChIP-seq (macrophages (GSM2663828) and RN2 cells (GSM2092897)) at *Csf1* and *Il6ra* loci. Grey rectangles encompass the region analysed for binding of ARID1A using ChIP-qPCR.

Supplementary Figure 11

a

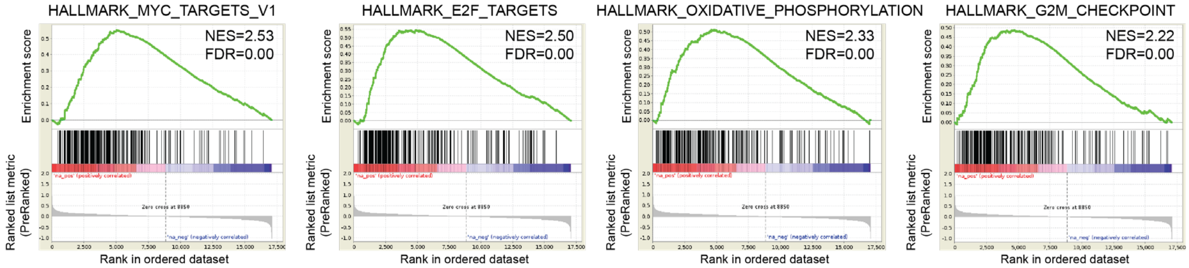

b

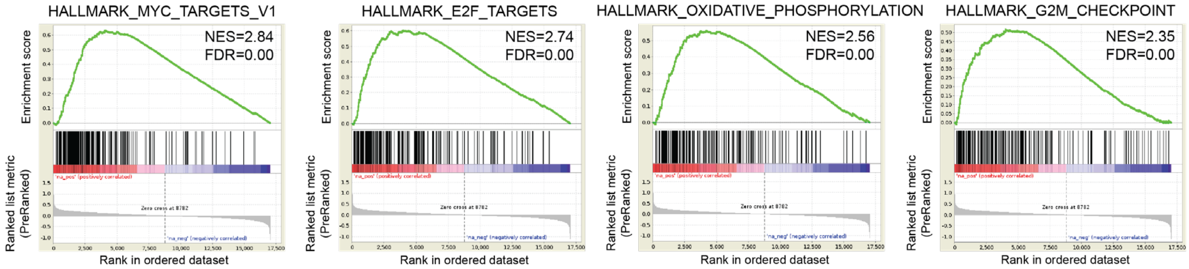

Supplementary Figure 11 (**a-b**) GSEA plots for top four enriched genesets in both *ARID1A* sg1 (a) and sg5-expressing (b) NB4 cells compared to the control cells. NES: Normalized enrichment score.

Supplementary Tables

Supplementary Table 1 Antibodies used for flow cytometric analysis

Supplementary Table 2 Primers used for quantitative RT-PCR and ChIP-PCR analysis

Supplementary Table 3 Frequency of pups of different genotypes from crosses of *Arid1a*$^{f/+}$*;Vav-iCre*$^{+}$ x *Arid1a*$^{f/+}$*;Vav-iCre*$^{-}$ mice

Supplementary Table 4 Differentially expressed genes in LT-HSCs of *Arid1a*$^{f/f}$*;Mx1Cre*$^{+}$ mice

Supplementary Table 5 Differentially expressed genes in CMP, GMP and MEP cells of *Arid1a*$^{f/f}$*;Mx1-Cre*$^{+}$ mice

Supplementary Table 6 ATAC-seq peaks significantly altered in *Arid1a* KO Lin^{-}Kit^{+} cells identified using DiffBind analysis

Supplementary Table 7 Genes differentially expressed in ARID1A-deficient NB4 cells

Separate excel sheets for Supplementary Tables 4-7

Supplementary Table 1 Antibodies used for flow cytometric analysis

No.	Name	Fluorochrome	Catalog Number	Clone	Supplier
1	CD117	APC	17-1171-83	2B8	eBioscience
2	CD117	APC-eFluo780	47-1171-82	2B8	eBioscience
3	CD11b	PE-Cy7	25-0112-82	M1/70	eBioscience
4	CD11b	FITC	11-0112-82	M1/70	eBioscience
5	CD127	AlexaFluor488	53-1271-82	A7R34	eBioscience
6	CD135	PE	12-1351-83	A2F10	eBioscience
7	CD150 (SLAM)	PE	115904	TC15-12F12.2	Biolegend
8	CD16/CD32	Biotin	45-0161-82	93	eBioscience
9	CD19	PE-Cy7	25-0193-82	eBio1D3	eBioscience
10	CD24	APC-eFluo780	47-0242-82	M1/69	eBioscience
11	CD25	AlexaFluor488	53-0251-82	PC61.5	eBioscience
12	CD34	FITC	11-0341-82	RAM34	eBioscience
13	CD3e	PE-Cy7	25-0031-82	145-2C11	eBioscience
14	CD3e	APC	17-0031-83	145-2C11	eBioscience
15	CD4	APC-eFluo780	47-0041-82	GK1.5	eBioscience
16	CD43	FITC	561856	S7	BDBioScience
17	CD44	PE-Cy7	25-0441-82	IM7	eBioscience
18	CD45.1	APC-eFluo780	47-0453-82	A20	eBioscience
19	CD45.2	FITC	11-0454-82	Co4	eBioscience
20	CD45R (B220)	PE-Cy7	25-0452-82	RA3-6B2	eBioscience
21	CD48	FITC	11-0481-82	HM48-1	eBioscience
22	CD71	APC	17-0711-82	R17217	eBioscience
23	CD8a	PE	12-0081-83	53-6.7	eBioscience
24	F4/80	APC	17-4801-82	BM8	eBioscience
25	IgM	APC	1020-118		Southern Biotech
26	Ly-51	PE	553735	BP-1	BDBioScience
27	Ly6A/E (Sca-1)	PerCP-Cy5.5	45-5981-82	P7	eBioscience
28	Ly6G (Gr-1)	PE-Cy7	25-5931-82	RB6-8C5	eBioscience
29	Ly-6G (Gr-1)	PE	12-5931-83	RB6-8C5	eBioscience
30	TER-119	PE-Cy7	25-5921-82	TER-119	eBioscience
31	CD11b	APC	17-0118-42	ICRF44	eBioscience

Supplementary Table 2 Primers used for quantitative RT-PCR and ChIP-PCR analysis

RT-PCR

Klf1 Forward	CCTCCATCAGTACACTCACC
Klf1 Reverse	CCTCCGATTTCAGACTCACG
Klf3 Forward	TGCAAGAGAACCATCCTTCC
Klf3 Reverse	GGTGCATTTGTACGGCTTTT
Gata2 Forward	GATACCCACCTATCCCTCCTATGTG
Gata2 Reverse	GTGGCACCACAGTTGACACACTC
Rxra Forward	GATATCAAGCCGCCACTAGG
Rxra Reverse	TGTTGTCTCGGCAGGTGTAG
Cebpa Forward	GACCATTAGCCTTGTGTGTACTGTATG
Cebpa Reverse	TGGATCGATTGTGCTTCAAGTT
Runx1 Forward	GCCACCTACCATAGAGCCATCAA
Runx1 Reverse	CCAATTCACTGAGCCGCTCGGAA
Csf1 Forward	GGGCCTCCTGTTCTACAAGT
Csf1 Reverse	GTTCCACCTGTCTGTCCTCA
Il6ra Forward	GGCACTCCTTGGATAGCAGA
Il6ra Reverse	GGGACATGGACGAGGATTCT
Csf3r Forward	TGTGTAGTGACCTGGCTCTG
Csf3r Reverse	CTGTCATGATGGTGGGCAAC
Cbfb Forward	GCACAACAGGCCTTTGAAGA
Cbfb Reverse	TCATCACCGCCACCTAAGTT
beta-actin Forward	GGCACCACACCTTCTACAATGAG
beta-actin Reverse	GGGTGTTGAAGGTCTCAAACATG

ChIP-qPCR

Cd34 Forward	GCCAAGATGACACACGGTTA
Cd34 Reverse	GGATATTGGCTCTCCTCCCC
Csf1 Forward	CTTGAGAGTTTCTACAGCTGCC
Csf1 Reverse	AGACAGCAACACAAGACAACAA
Gata2 Forward	AGACAGCCAGAACCAAGAGAG
Gata2 Reverse	TTGAGGTGATCTTAGGCCAGG
Il6ra Forward	CCAGGTTTTGACGCTGAGATAA
Il6ra Reverse	CATGAAAGCAAAACTCAAGCGG
Cebpa Forward	CACGATCTCTCTCCACTAGCA
Cebpa Reverse	CGCTTTTATAGAGGGTCGGGC

Supplementary Table 3 Frequency of pups of different genotypes from crosses of *Arid1a*$^{f/+}$*;Vav-iCre*$^{+}$ x *Arid1a*$^{f/+}$*;Vav-iCre*$^{-}$ mice

**Genotype**	**Cre+**			**Cre-**		
	f/f	f/+	+/+	f/f	f/+	+/+
**Expected frequency**	12.5%	25.0%	12.5%	12.5%	25.0%	12.5%
**Weaning (n=179)**	2.8% (5)	26.8% (48)	17.3% (31)	12.3% (22)	26.8% (48)	14.0% (25)
**Newborn (n=89)**	7.9% (7)	28.1% (25)	14.6% (13)	13.5% (12)	24.7% (22)	11.2% (10)

Number of pups are indicated in parentheses
